# The evolving literature on the ethics of artificial intelligence for healthcare: a PRISMA scoping review

**DOI:** 10.3389/fdgth.2025.1701419

**Published:** 2025-11-20

**Authors:** Yufei Wang, Alex Federman, Heather Wurtz, Margaret A. Manchester, Lillian Morgado, Catherine E. A. Scipion, Maria Adjini, Kendall Williams, Benjamin Wills, Victoria Helmly, Jalayne J. Arias

**Affiliations:** 1 Department of Health Policy and Behavioral Sciences, School of Public Health, Georgia State University, Atlanta, GA, United States; 2 Division of General Internal Medicine, Icahn School of Medicine at Mount Sinai, New York, NY, United States; 3 Department of Population and Public Health Sciences, Keck School of Medicine, University of Southern California, Los Angeles, CA, United States; 4 Department of Sociology and Science Studies Program UC San Diego, La Jolla, CA, United States

**Keywords:** artificial intelligence, ethics, policy and legal issues, healthcare, scoping review

## Abstract

This scoping review analyzes the literature on the ethics of artificial intelligence (AI) tools in healthcare to identify trends across populations and shifts in published research between 2020 and 2024. We conducted a PRISMA scoping review using structured searches in PubMed and Web of Science for articles published from 2020 to 2024. After removing duplicates, the study team screened all sources at three levels for eligibility (title, abstract, and full text). We extracted data from sources using a Qualtrics questionnaire. We conducted data cleaning and descriptive statistical analyses using R version 4.3.1. A total of 309 sources were included in the analysis. While most sources were conceptual articles, the number of empirical studies increased over time. Commonly addressed ethical concerns included bias, transparency, justice, accountability, privacy/confidentiality, and autonomy. In contrast, disclosure of AI-generated results to patients was infrequently addressed. There was no clear trend indicating greater attention to this topic within our period of review. Among all eligible sources, the proportion addressing legal and policy issues broadly has shown a declining trend in recent years. There was an uptick in the number of sources discussing legal liability, patient acceptability, and clinician acceptability. Yet, these three topics remained infrequently addressed overall. Significant gaps in research on the ethics of AI applications in healthcare include disclosure of results to patients, legal liability, and patient and clinician acceptability. Future research should focus more on these ethical issues to facilitate the responsible and appropriate implementation of AI in healthcare.

## Introduction

Artificial intelligence (AI) is transforming the landscape of healthcare by enhancing prevention, diagnosis, and treatment across a broad spectrum of medical conditions (1). The potential value of AI healthcare applications (AI-HCA) is immense, including lowering healthcare costs and improving care delivery (2). However, the advancement of AI methods for clinical applications has introduced novel ethical challenges (e.g., concerns about bias and transparency) that may impede clinical adoption. Many of these issues have been explored in the biomedical ethics literature, which has expanded substantially in recent years (3–5). The growing body of literature at the intersections of AI-HCA and ethics has touched on a wide range of themes and used various methodologies, resulting in uncertainty about its clinical and policy implications and future directions for research. Identifying common themes through the published work could inform solutions that resolve ethical dilemmas and support responsible and appropriate implementation of AI-HCA.

To inform future research and scholarship on the ethics of AI-HCA, we conducted a scoping review and synthesis of emerging norms in recent ethics literature. Specifically, we sought to identify the ethical norms that have emerged, and to examine where within the literature there is consensus, dissension, and gaps regarding ethical challenges that need resolution.

## Materials and methods

### Study design

This study followed the Preferred Reporting Items for Systematic reviews and Meta-Analyses extension for Scoping Reviews (PRISMA-ScR) (6) to map existing ethics literature regarding AI-HCA. We developed a PRISMA-ScR protocol prior to the review to guide our methodology, ensuring transparency and methodological rigor throughout the process.

### Search strategy

We developed structured search terms (Supplementary File S1) tailored to each platform aimed at capturing publications that would address our research questions. We conducted two searches at different time points. Both searches were conducted in PubMed and Web of Science. The initial search in February 2023 included sources from 2020 to 2022. Given the rapid growth of the field, we repeated the search in December 2024 to include literature published between 2023 and 2024. Microsoft Excel was utilized for organization of titles, abstracts, and preliminary review.

### Eligibility criteria

Eligibility criteria included: (1) publications dated published between 2020 and 2024, (2) published in English, (3) publication types of interest (conceptual articles, white papers, formal reviews, empirical research, or guideline/consensus statement), and (4) relevant to AI (e.g., machine learning) applications in healthcare settings. Additional details of the terminology input into the search engines may be found in the appendix.

### Source selection

Duplicate sources were removed. Three rounds of review were then used to refine the sample. Eligibility was assessed by title in the first round, abstract in the second round, and full text in the third round. Each source was screened independently by two team members for eligibility in the first two rounds. Results were compared and divergent codes were reviewed and agreed upon during consensus meetings. The third round of review was conducted during the full text analysis while extracting data from the article. A senior team member (JA) reviewed all articles deemed ineligible at the full-text analysis stage. All reviewers ultimately agreed with the final decisions on the articles that initially received conflicting assessments from different reviewers. Only articles eligible after all three rounds were included in the analysis.

### Data extraction

Consistent with study objectives and overarching research questions, we designed a questionnaire in Qualtrics (Qualtrics Company, US) to collect and analyze relevant domains (Supplementary File S2). The first domain of the questionnaire was used to collect basic details (authors, title, journal, year of publication) and the type of study (empirical or conceptual) for each source. The second domain included details reported within the article regarding the AI technology or concept described within the source. This also included the notation of clinical AI-specific methods employed (machine learning, general AI, or others), and whether an AI tool was in use or hypothetical. In the third domain, we collected data on ethical norms, legal topics, and policy topics reported within the source (see Supplementary File S2 for specificity).

### Data analysis

We exported data from Qualtrics to R studio for cleaning and descriptive statistics reporting, including the frequency and rates of topics by year. All analyses were conducted using R version 4.3.1.

## Results

### Literature search

**Figure 1:**
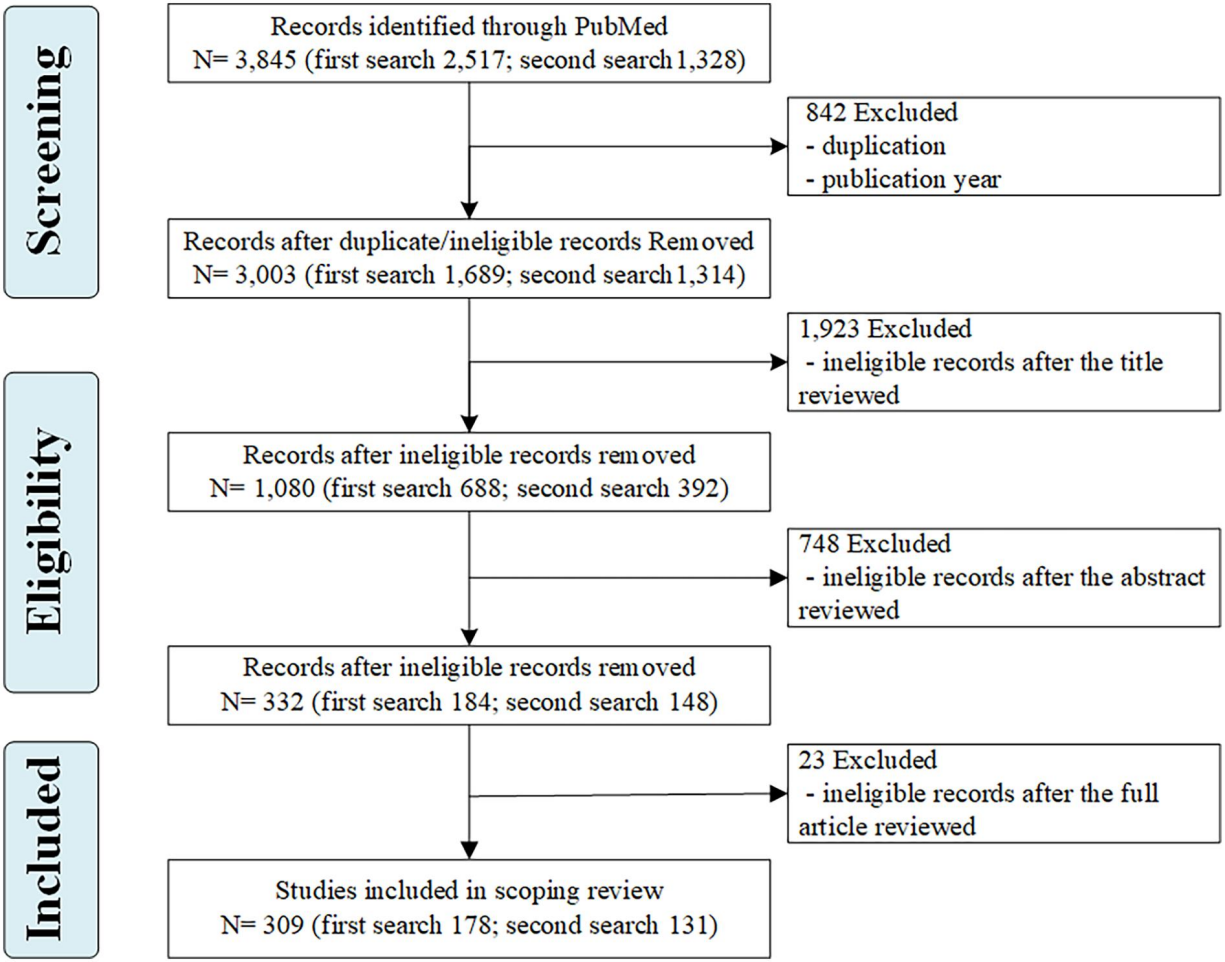
Flow chart of the selection process.

The first search yielded 2,517 sources and the second yielded 1,328 sources (Figure 1). After removing duplicates and sources that were not eligible based on publication date, a total of 309 sources (Supplementary Table 1) were included for full analysis.

### Characteristics of the included studies

**Figure 2:**
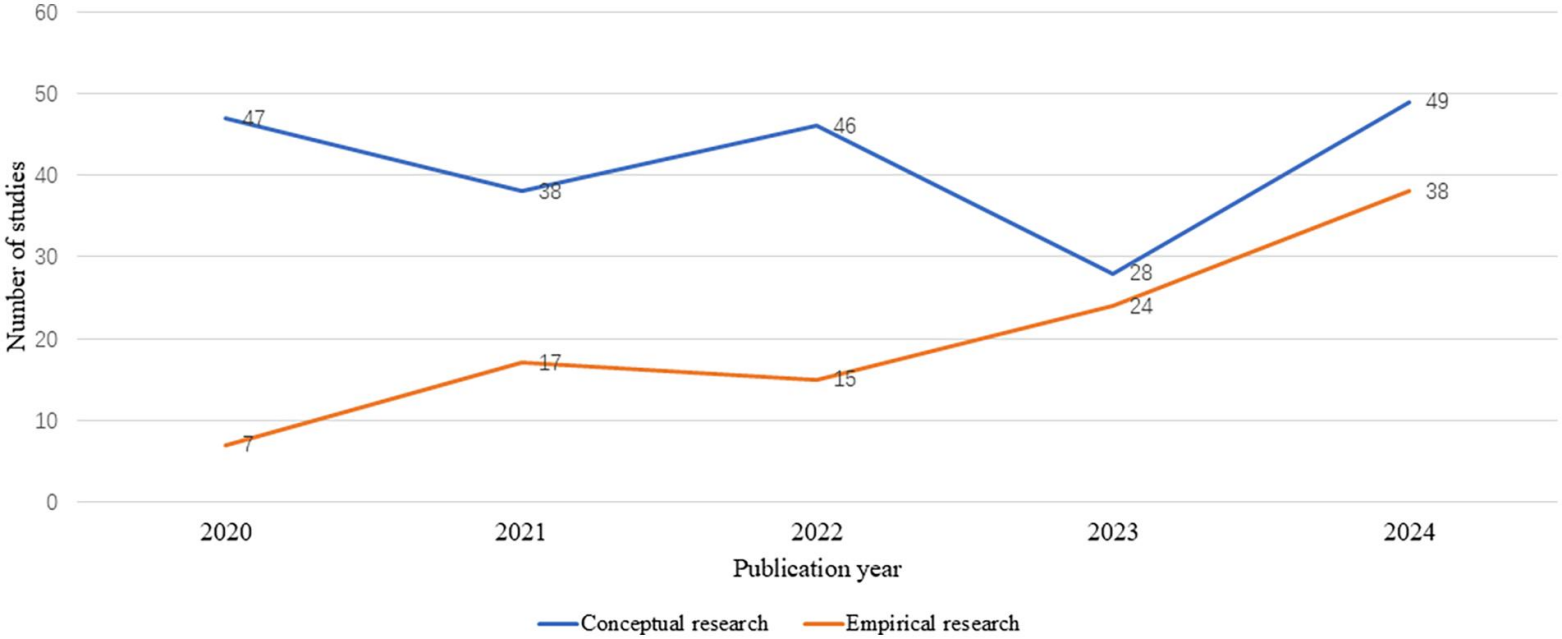
Trends in article type by year.

Among the 309 eligible sources, we observed a linear increase in the number of papers published from 2020 to 2024, except for a lower frequency in 2023 (Table 1). Among the sources analyzed, 67.3% were conceptual while 32.7% were empirical. Additionally, conceptual sources outnumbered empirical research each year from 2020 to 2024, while the number of empirical sources increased over time (Figure 2). Among empirical sources, a majority were structured reviews (formal meta-analysis or other literature review) (56.4%), followed by qualitative (interviews, focus groups) (27.7%), survey (9.9%), and other (5.9%). Regarding the evaluation of AI tools, 47.9% of sources evaluated hypothetical AI tools (i.e., tools not currently in use). The literature reflected ethical analysis of AI-HCA for diverse clinical uses, with most sources evaluating tools with no specific clinical applications (57.9%), followed by diagnosis (46.9%), treatment (31.1%), clinical monitoring (23.9%), and screening (21.4%).

**Table 1:** Characteristics of the included studies (*N* = 309).

Characteristics	*N* (%)
Publication year	
2020	54 (17.5%)
2021	55 (17.8%)
2022	61 (19.7%)
2023	52 (16.8%)
2024	87 (28.2%)
Article type	
Empirical	101 (32.7%)
Conceptual	208 (67.3%)
Methods used in empirical research^a^	
Review (formal meta or other literature review)	57 (56.4%)
Qualitative (interviews, focus groups)	28 (27.7%)
Survey	10 (9.9%)
Other	6 (5.9%)
AI tool	
In use	84 (27.2%)
Hypothetical	148 (47.9%)
Unsure/Not specified	77 (24.9%)
Clinical use of the AI tool^b^	
Other/Non-specific	179 (57.9%)
Diagnostic	145 (46.9%)
Treatment	96 (31.1%)
Clinical monitoring	74 (23.9%)
Screening	66 (21.4%)

a The proportion was calculated by n/101*100% since there were 101 empirical articles.

b The proportion was calculated as n/309*100% due to the “select all that apply” question format.

### Frequency of ethical issues raised or evaluated in literature

**Figure 3:**
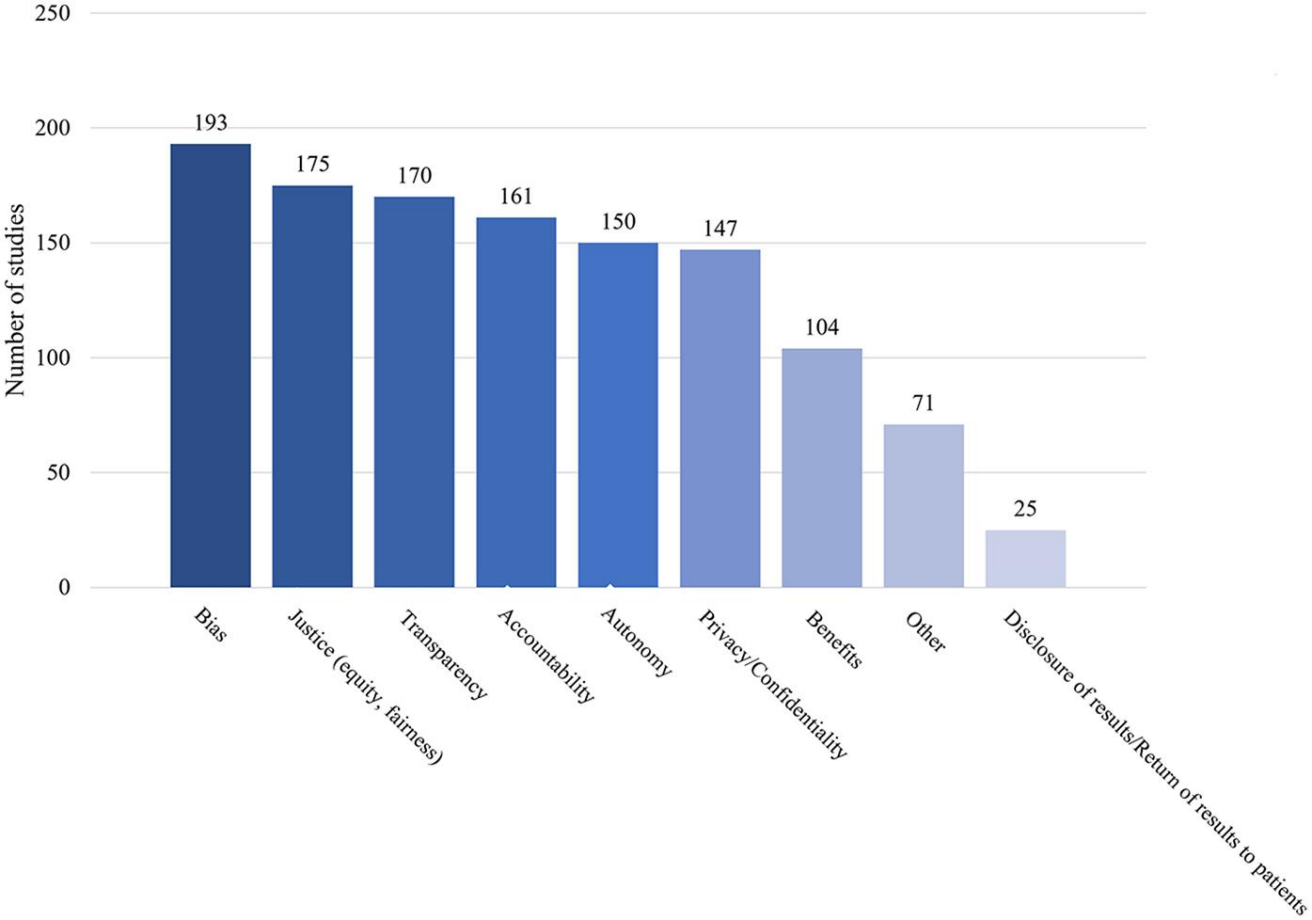
Ethical issues raised in the sources.

Our results revealed patterns in the frequency with which ethical norms are addressed across sources (Figure 3). Bias arising from under-representative datasets or algorithms powering AI-HCA emerged as the most frequently addressed ethical issue, in 62.5% of sources. Yoon et al. (7) demonstrated one example (7). In their article, a proprietary healthcare insurance algorithm used health costs as a proxy for health needs and was given accurate information that Black Americans had less access to healthcare. This resulted in an AI algorithm that produced biased results, with a >50% reduction in the number of Black patients identified for extra care (8). Other frequently identified ethical issues included justice (56.6% of sources) and transparency (55.0% of sources). Justice was most often related to fair access of AI-HCA benefits across populations. Sources that included transparency as a topic addressed openness and clarity concerns of how AI systems function and deliver clinical decisions or recommendations. Comparatively, “disclosure of results to patients” (8.1%) was rarely referenced in the sources. This was used to identify references to all healthcare-related results, including AI involvement in clinician decision-making.

**Figure 4:**
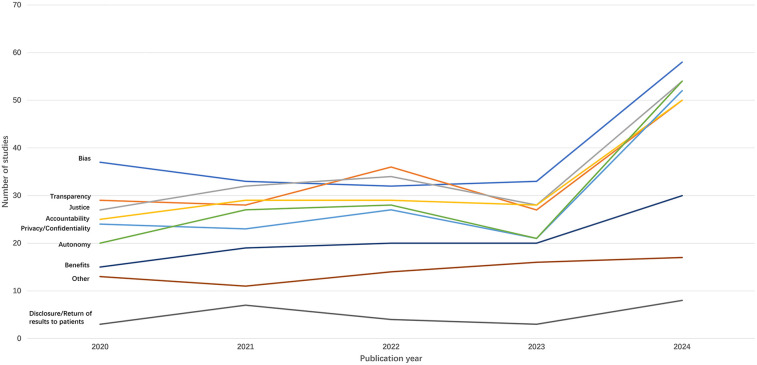
Trends in the number of sources addressing ethical issues by year.

**Figure 5:**
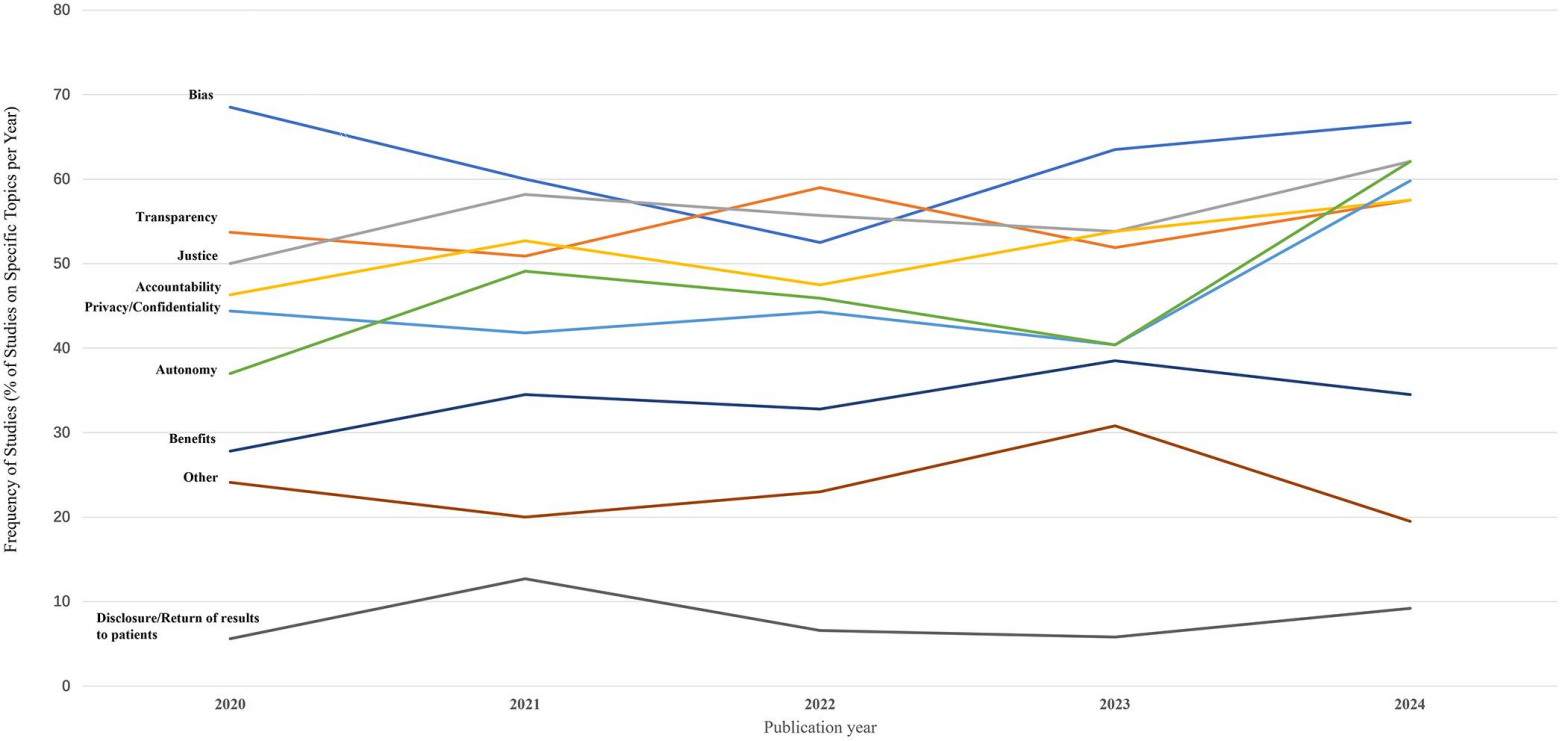
Trends in the proportion of sources addressing specific ethical issues by year.

Frequency and rate of topic varied across years (Figures 4, 5). Transparency, justice, accountability, privacy/confidentiality, and autonomy all surged in both number and rate in 2024, consistent with the overall trend of the highest number of sources appearing in 2024. The absolute number of sources addressing bias peaked in 2024 (*N* = 58), while the proportion of such sources declined from 2020 through 2022, then rose again. Benefits of AI-HCA, disclosure or return of results to patients, and other topics (e.g., clinical validation, the human-like aspects of GenAI, ownership, and data rights) were consistently the least commonly addressed ethical topics each year from 2020 to 2024. Nonetheless, sources increasingly addressed the benefits of AI-HCA throughout the 5-years of literature reviewed.

### Frequency of legal & policy issues evaluated in literature

**Figure 6:**
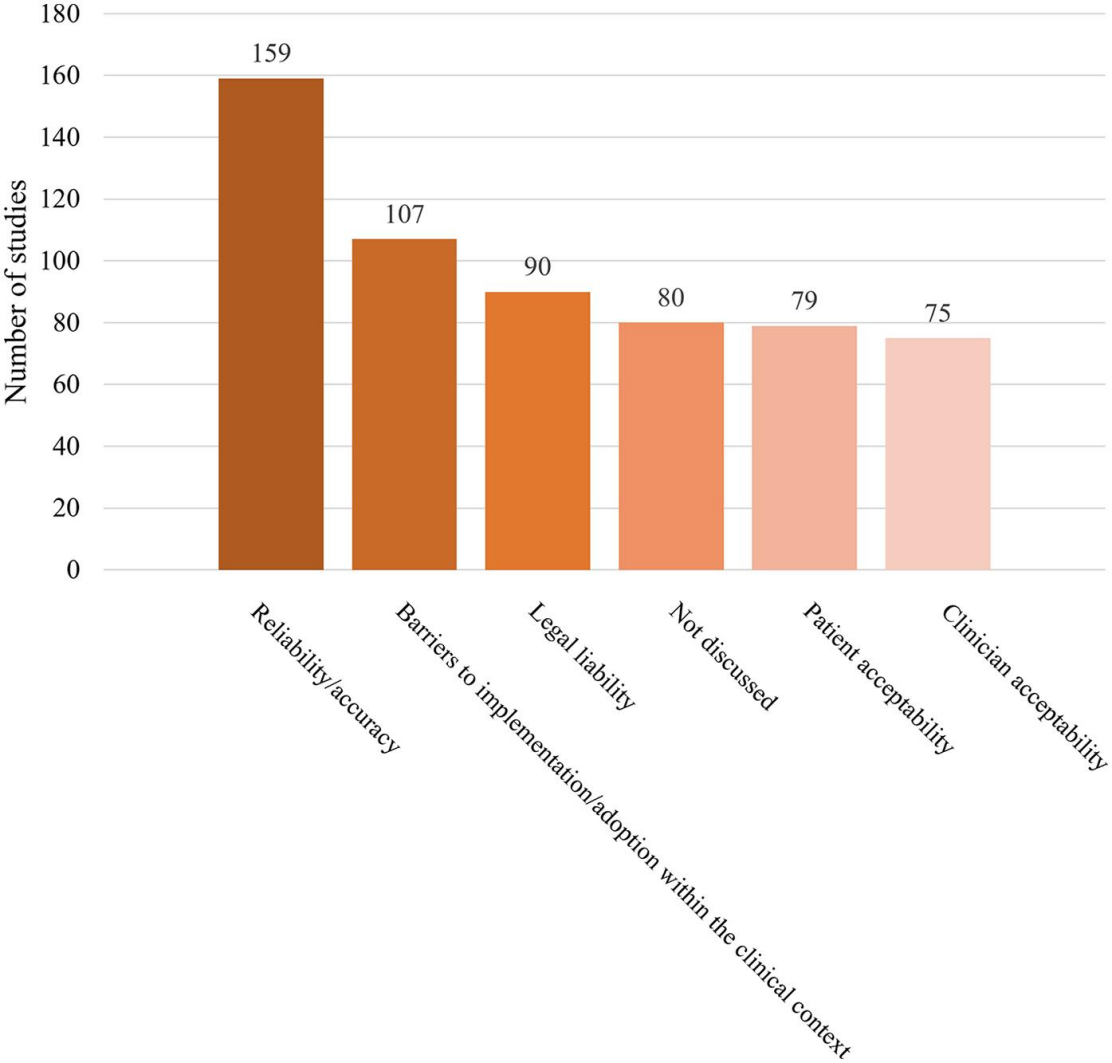
Legal or policy topics raised in the sources. Note: “Not discussed” refers to the sources that did not address any of the following topics: reliability/ accuracy, barriers to implementation/adoption, legal liability, patient acceptability, or clinician acceptability.

In addition to the ethical issues, we also extracted data on legal and policy issues that might be informative. In this context, legal issues represented specific legal doctrine (e.g., liability). Comparatively, policy issues were broader considerations and themes that might inform diverse forms of policy setting. Among legal and policy issues evaluated in the literature (Figure 6), reliability and accuracy were the most frequent (51.5%). Reliability/accuracy was identified as the performance precision of applied AI tools. Barriers to implementation/adoption, patient acceptability, and clinician acceptability were less frequently mentioned in the sources, at 107 (34.6%), 79 (25.6%), and 75 (24.3%), respectively. Sources that assessed barriers to implementation/adoption frequently referenced challenges such as difficulties translating tools developed by AI professionals into clinical practice, a lack of robust and integrated AI data sources, and inadequate trust (in AI adoption) among stakeholders (e.g., clinicians, patients). This was attributed to concerns regarding interpretability and explainability, commonly referred to as the “black box” issue.

Legal liability was referenced in nearly one-third of the sources (*n* = 90, 29.1%). “Legal liability” includes responsibility (legally) for AI-tool caused harm (e.g., misdiagnosis). Some articles argued that physicians should be held accountable and liable for clinical decisions aided by information provided by AI tools. This subset of literature recommended that health professionals should not blindly follow suggestions from AI, they could use AI as a confirmatory tool to support clinical decision-making. In this way, they can reduce the risk of legal repercussions associated with AI-guided decisions (1).

**Figure 7:**
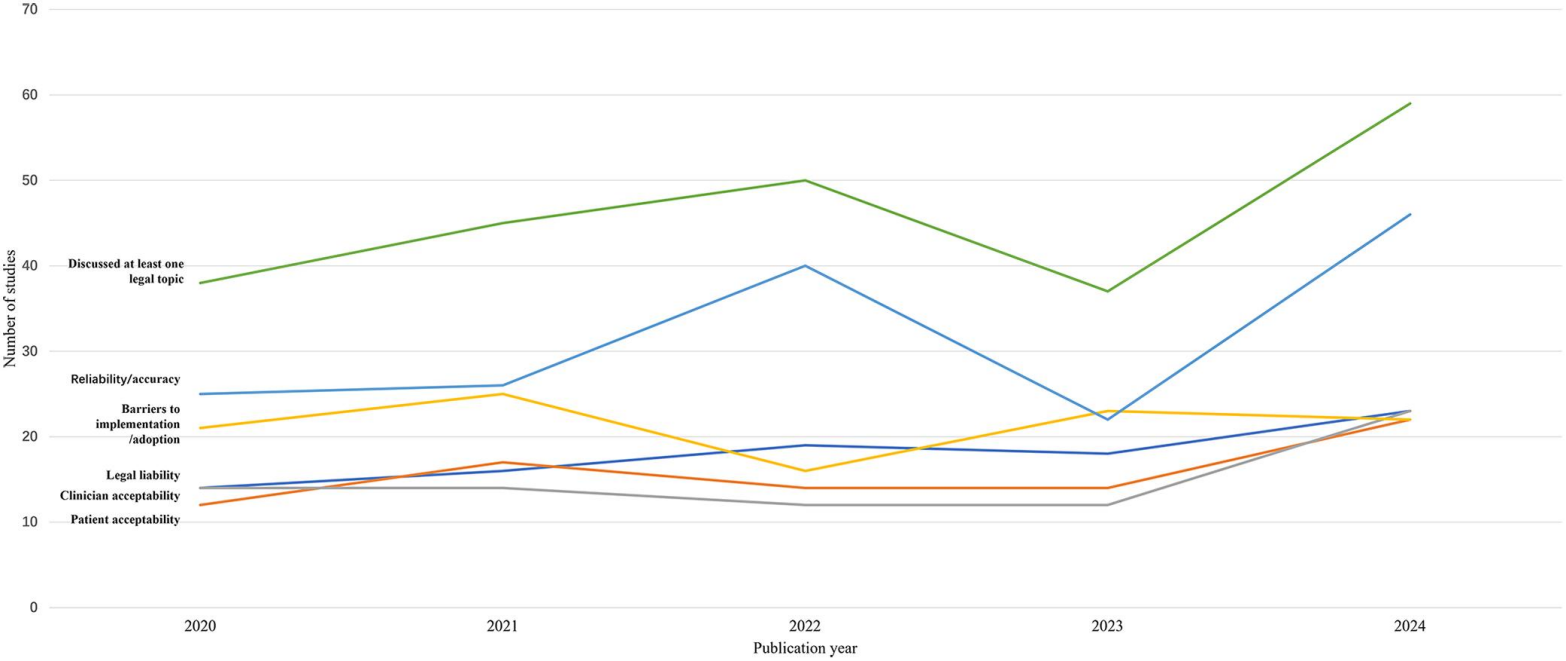
Trends in the number of sources addressing legal/policy topics by year.

**Figure 8:**
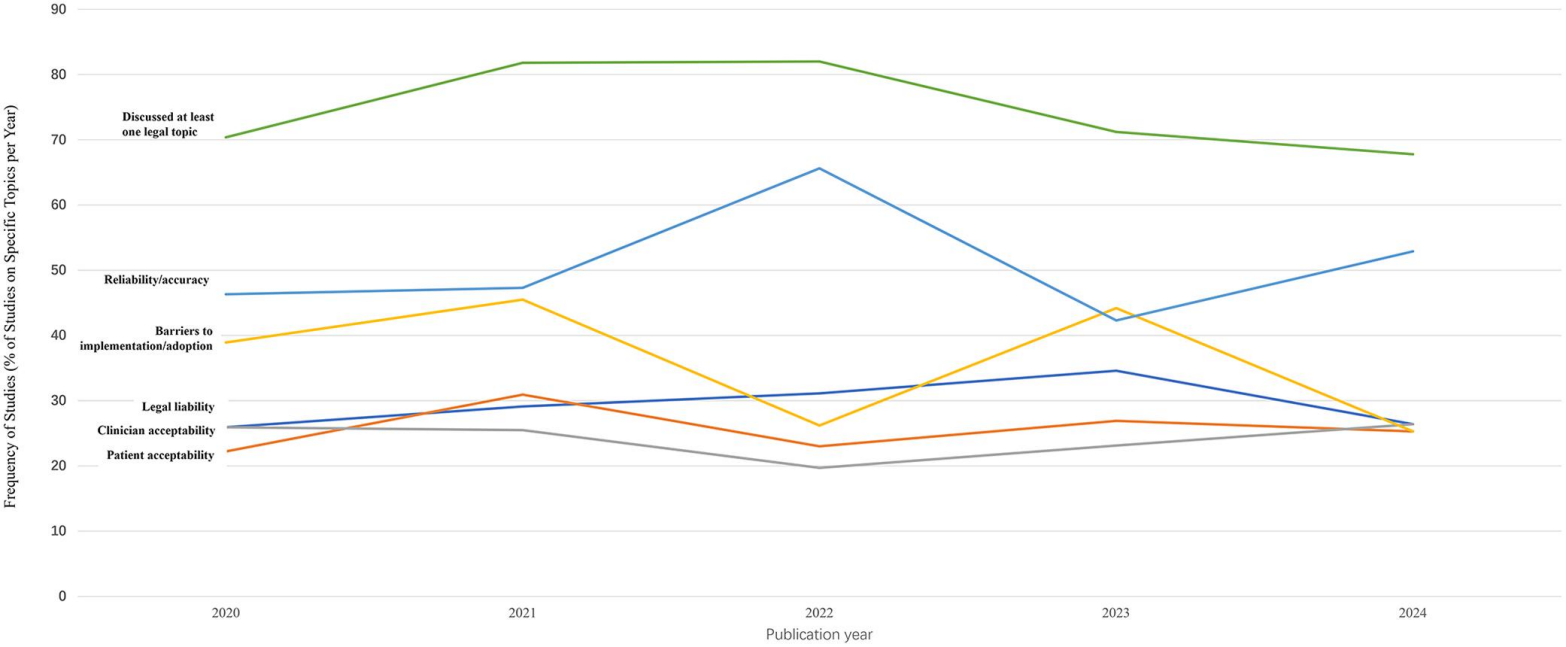
Trends in the proportion of sources addressing specific legal/policy topic by year.

We explored the trends in legal/policy codes from 2020 to 2024 (Figures 7, 8). Although the number of sources that evaluated legal or policy topics increased substantially in 2024 (with 38, 45, 50, 37, and 59 sources from 2020 to 2024, respectively), the proportion of sources addressing legal or policy topics showed a general downward trend from 2020 to 2024 (with 70.4%, 81.8%, 82%, 71.2%, 67.8%, respectively). Literature that included a focus on specific codes also had a significant fluctuation in reliability/accuracy over time. In contrast, barriers to implementation/ adoption, legal liability, patient acceptability, and clinician acceptability were mentioned less frequently in sources and were similarly prevalent in 2024.

## Discussion

This study evaluated 309 sources on AI-HCA ethics published between January 2020 and December 2024 using a PRISMA-ScR. While a majority of the published sources reflected conceptual work, we found that the number of published empirical studies increased between 2020 and 2024. Diagnosis was the most common clinical application for AI tools. Bias, transparency, justice, accountability, privacy/confidentiality, and autonomy were the frequently evaluated ethical concerns. Benefits were infrequently addressed, but it became an increasingly frequent topic in literature over time. In contrast, the least discussed ethical norm was disclosing to patients that results are produced or aided by AI. Among all eligible sources, the proportion that addressed legal and policy considerations broadly has shown a declining trend in recent years.

Ethical concerns about AI-HCA vary across studies. Due to the “black box” nature of AI, transparency, explainability, and the trust of both patients and healthcare professionals were frequently raised as ethical concerns in some research (9–12). The public has doubts about the accuracy and reliability of health data recorded by AI because of limited transparency and explainability (12, 13). To mitigate the negative effects of these limitations, several methods have been proposed, including disclosing the nature and sources of the data used by AI, proposing explainable AI, and ensuring the knowability of the AI system's purposes (11, 14). A few studies highlighted accountability and who bears the responsibility for harm. There is a lack of consensus in the literature and it remains unclear what factors physicians should consider when making clinical decisions with AI assistance, and who is accountable for AI's actions when things go wrong (15, 16). Additionally, some studies argued that interpretability was crucial, as it can help address and minimize biases, with significant implications for patients' autonomy, racial equity, scientific reproducibility, and generalizability (17, 18). The uncertain accountability, unexplainable nature of AI systems, lack of interpretability, and other ethical issues pose a significant threat to the successful implementation of AI in clinical settings.

### Ethical standards/norms

Although the trend in the quantity and proportion of sources addressing bias has fluctuated, it remains the most frequently appearing code in recently published sources. This finding is consistent with the results of other studies (19, 20). The significant attention given to bias by researchers is understandable, as bias is an inherent risk of AI. As Friedrich et al. proposed, since humans design algorithms, AI systems may inevitably reflect human prejudice, misunderstanding, and bias (21). Numerous studies have noted that deliberate investments are necessary to minimize bias and ultimately improve the trustworthiness of AI-HCA and mitigate health disparities (14, 22).

Transparency was frequently mentioned in the studies we reviewed. Previous research stated that transparency poses a significant challenge in cases where AI employs “black box” algorithms (23–25). These algorithms often stem from complex machine learning and neural network models that are difficult for clinicians to fully comprehend. Questions such as, “To what extent does a clinician need to disclose that they cannot fully interpret the diagnosis or treatment recommendations provided by the AI?” and “How much transparency is needed?” cannot be answered at this time (26).

The current study found that discussions of justice, accountability, autonomy, and benefit are becoming increasingly common, with more sources addressing these topics over time, especially in 2024. For instance, Tahri et al. noted that promoting accountability is fundamental to ensure that AI is used to develop better healthcare systems (27). The trend was implied by previous studies (22, 28, 29) that also highlighted the significance of these ethical norms. In contrast, the topic about disclosure of results to patients was the least mentioned in sources included in the current study, which is contrary to the expectation that research in the evolving field of AI in healthcare should increasingly incorporate patient perspectives into the standards used in practice (30). The finding of limited research on disclosing results to patients was consistent with previous studies. As Rose and Shapiro noted, the limited studies on patients' perspectives regarding the use of AI in healthcare make it difficult to draw generalizable conclusions (30).

The use of AI in healthcare requires vast amounts of sensitive patient data. Elendu and colleagues argued that ensuring the privacy and security of this data was paramount, as any breach could severely impact patient trust and data integrity (20). However, our study revealed that fewer than half of the sources addressed privacy or confidentiality, and there is no clear trend of increasing interest in the topic. Future research may need to place greater emphasis on privacy/confidentiality.

### Legal or policy topics

There has been a declining trend in the proportion of sources evaluating legal and policy considerations in recent years. However, integrating law, policy, and ethics is likely essential for society to fully reap the benefits of medical innovation while avoiding its potential pitfalls during dissemination (31).

Reliability/accuracy, the most frequently mentioned issue in studies we reviewed, were prominent topics in previous research (31). Legal liability, patient acceptability, and clinician acceptability were discussed less frequently in the reviewed studies, and there was no clear trend indicating that these topics received increased attention in ethical AI-HCA sources over time. These results align with Martinho et al.'s finding that there is limited knowledge regarding the perspectives of medical doctors on the ethical issues associated with the implementation of AI in healthcare (32). Patient acceptability and clinician acceptability should receive more attention if we aim to ensure that AI for health is designed and used in a manner that respects human dignity, fundamental rights, and values.

### Limitations

This scoping review provides important insights into existing gaps in the literature and evolving trends regarding the ethical evaluation of AI-HCA. Several limitations should be noted. First, we searched only PubMed and Web of Science, and there may be relevant sources published in other databases that were not included in our analysis. Therefore, we cannot rule out the possibility that a more extensive search could have yielded different results. Second, we included only sources published between January 2020 and December 2024. While this short timeframe allows for a focused analysis of trends in the popularity of ethical and legal topics, it limits our ability to observe longer-term trends of ethical norms and legal issues. Third, we restricted our search to English-language sources, which may have prevented us from getting a full picture of the focus or tendency of AI-HCA ethics.

## Conclusions

Although empirical research on ethical issues related to AI in clinical settings has increased over time, conceptual research remains the predominant form. As a result, a paucity of empirically driven research at the intersection of ethics and AI-HCA limits guidance that is needed to support adoption of these tools clinically. Conceptual work provides an important defining and framing the normative considerations that may affect clinical adoption. However, empirical data is needed to better isolate and prioritize ethical challenges and to generate solutions to mitigate potential barriers. This is particularly true for ethical topics that receive less attention within literature (e.g., the disclosure of AI-tool generated results) and those that may be specific to clinical implementation (e.g., autonomy/consent models and privacy/confidentiality). AI-HCA have immense potential to alter and benefit clinical practices. However, gaps in existing research on ethical issues that could impede adoption must be addressed to harness these benefits.

## Data Availability

The original contributions presented in the study are included in the article/Supplementary Material, further inquiries can be directed to the corresponding author.
