# Supplementary material for: The evolving literature on the ethics of artificial intelligence for healthcare: a PRISMA scoping review
Source: Front Digit Health. 2025 Nov 20;7:1701419. doi: 10.3389/fdgth.2025.1701419 (PMC12675450; doi:10.3389/fdgth.2025.1701419)
Supplement: Supplementary file 1 [file Supplementaryfile1.pdf]

S1 File. Detailed literature search process

Specifically, the search terms for PubMed were: (machine learning[mh] OR machine learning[tiab] OR artificial intelligence[mh] OR artificial intelligence[tiab] OR machine intelligence[tiab] OR computer reasoning[tiab]) AND ((morals[mh] OR morals[tiab] OR ethics[mh] OR ethics[tiab]) OR (ethics[sh])) AND (eng[la]) AND ((2000/01/01[CRDT] : 2030/12/31[CRDT]) OR (2000/01/01[EDAT] : 2030/12/31[EDAT]) OR (2000/01/01[MHDA] : 2030/12/31[MHDA])).

The search terms for Web of Science were: ((TS=(machine learning OR artificial intelligence OR computer reasoning) OR TI=(machine learning OR artificial intelligence OR computer reasoning) OR AB=(machine learning OR artificial intelligence OR computer reasoning)) AND (TS=(morals OR ethics) OR TI=(morals OR ethics) OR AB=(morals OR ethics)) AND (TS=(framework OR model OR guid*)) AND (LA=(English))) AND (DOP=(2000-01-01/2030-12-31))
