# Supplementary material for: The evolving literature on the ethics of artificial intelligence for healthcare: a PRISMA scoping review
Source: Front Digit Health. 2025 Nov 20;7:1701419. doi: 10.3389/fdgth.2025.1701419 (PMC12675450; doi:10.3389/fdgth.2025.1701419)
Supplement: Supplementary file 2 [file Supplementaryfile2.pdf]

Default Question Block

Authors (last, first initial)

Article title

Journal

Year published

Is this article eligible for our study? (Eligible studies evaluate, consider, or discuss the ethical issues associated with clinical use of AI)

- Yes
- No

If not eligible, please describe why?

Was this article reporting empirical work or a conceptual analysis?

- Empirical
- Conceptual

If empirical research, what methods were used in the reported article?

- ○ Survey
- ○ Qualitative (interviews, focus groups)
- ○ Review (formal meta or other literature review)
- ○ Other

Does the article evaluate an AI tool or method that was IN USE at the time of the article? (e.g., are the authors evaluating actual experience or hypothetical adoption).

- ○ Yes, the article is evaluating a tool or method that was in use
- ○ Hypothetical
- ○ Unsure/Not specified

What AI method/tool was considered/evaluated?

- ○ Machine Learning
- ○ AI Generally (no specific method used)
- ○ Other

If other please describe:

Does the paper evaluate a language based tool (e.g., natural language processing, textual analysis)

- ○ Yes
- ○ Does not evaluate it specifically - but could be included within the evaluation
- ○ No - evaluates a different form of analysis (e.g., generative tool or other non language algorithm)

What is the clinical use of the AI method/tool (Select all that apply)?

- ☐ Screening
- ☐ Diagnostic
- ☐ Clinical monitoring
- ☐ Treatment
- ☐ Other/Non-specific

What ethical standards/norms are raised in the evaluation? (Select all that apply)

- ☐ Autonomy (including consent)
- ☐ Justice (equity, fairness)
- ☐ Bias
- ☐ Benefits
- ☐ Accountability
- ☐ Privacy/Confidentiality
- ☐ Transparency
- ☐ Disclosure of results/Return of results to patients
- ☐ Other

Describe ethical standards/norms raised:

Did the article discuss any of the following? Select those discussed, select not discussed if none were referenced:

- ☐ Accountability (e.g., who is ethically responsible for false results)

- ☐ Legal liability
- ☐ Patient acceptability
- ☐ Clinician acceptability
- ☐ Barriers to implementation/adoption within the clinical context
- ☐ Reliability/accuracy
- ☐ Not discussed

If discussed, please describe here:

Please include any other notable details about this article

Powered by Qualtrics
