# Supplementary material for: The evolving literature on the ethics of artificial intelligence for healthcare: a PRISMA scoping review
Source: Front Digit Health. 2025 Nov 20;7:1701419. doi: 10.3389/fdgth.2025.1701419 (PMC12675450; doi:10.3389/fdgth.2025.1701419)
Supplement: Supplementary file 3 [file Table1.docx]

S1 Table. Summary of Literature Review Table

| **Authors** | **Year** | **Article type** | **AI type** | **Clinical use** | **Ethical standards/norms** | **Legal or policy topics** |
| --- | --- | --- | --- | --- | --- | --- |
| Cobianchi L, Verde JM, Loftus TJ, Piccolo D, Dal Mas F, Mascagni P, Garcia Vazquez A, Ansaloni L, Marseglia GR, Massaro M, Gallix B, Padoy N, Peter A, Kaafarani HM | 2022 | Conceptual | AI Generally (no specific method used) | Other/Non-specific | Autonomy (including consent),Justice (equity or fairness),Bias,Benefits,Accountability (ethically responsible for results),Privacy/Confidentiality,Transparency,Other | Legal liability,Reliability/accuracy, |
| Friedrich, A.; Mason, J.; Malone, J. | 2022 | Conceptual | AI Generally (no specific method used) | Other/Non-specific | Bias,Other | Reliability/accuracy |
| Aquino, Y., Rogers, W., Braunack-Mayer, A., et al | 2023 | Empirical | AI Generally (no specific method used) | Diagnostic | Other | Clinician acceptability, |
| Rathkopf C., Heinrichs B. | 2023 | Conceptual | AI Generally (no specific method used) | Other/Non-specific | Other | Not discussed |
| Bear Don't Walk IV O., Reyes Nieva H, Lee S., Elhadad N. | 2022 | Empirical | Machine Learning | Other/Non-specific | Autonomy (including consent),Justice (equity or fairness),Bias,Privacy/Confidentiality | Not discussed |
| Beltramin D, Lamas E, and Bousquet C | 2022 | Conceptual | Other | Screening,Diagnostic,Other/Non-specific | Justice (equity or fairness),Bias,Benefits,Transparency | Patient acceptability,Reliability/accuracy, |
| Cao X., Liu X. | 2022 | Conceptual | AI Generally (no specific method used) | Screening | Autonomy (including consent),Justice (equity or fairness),Benefits,Privacy/Confidentiality,Other | Barriers to implementation/adoption within the clinical context |
| Holm, S. | 2022 | Conceptual | AI Generally (no specific method used) | Screening,Diagnostic | Justice (equity or fairness) | Reliability/accuracy |
| Hatherley, J., Sparrow, R., Howard, M. | 2022 | Conceptual | AI Generally (no specific method used) | Diagnostic | Accountability (ethically responsible for results) | Reliability/accuracy |
| Kumar, P., Chauhan, S., Awasthi, LK. | 2023 | Empirical | AI Generally (no specific method used) | Diagnostic,Treatment | Accountability (ethically responsible for results),Privacy/Confidentiality | Legal liability |
| Pozzi G. | 2023 | Conceptual | Machine Learning | Other/Non-specific | Autonomy (including consent),Justice (equity or fairness),Bias | Not discussed |
| Jongsma K. R., Sand M. | 2022 | Conceptual | AI Generally (no specific method used) | Diagnostic | Accountability (ethically responsible for results),Transparency | Reliability/accuracy |
| Kempt H., Nagel S. K. | 2022 | Conceptual | Other | Diagnostic | Accountability (ethically responsible for results),Transparency | Legal liability,Reliability/accuracy |
| Goisauf, M., Abadia, M. | 2022 | Empirical | AI Generally (no specific method used) | Screening,Diagnostic,Clinical monitoring,Treatment | Autonomy (including consent),Justice (equity or fairness),Bias,Accountability (ethically responsible for results),Privacy/Confidentiality,Transparency,Other | Legal liability,Patient acceptability,Clinician acceptability,Barriers to implementation/adoption within the clinical context,Reliability/accuracy |
| Vandemeulebroucke, T; Denier, Y; Gastmans, C | 2022 | Empirical | Machine Learning | Other/Non-specific | Autonomy (including consent),Justice (equity or fairness),Privacy/Confidentiality | Not discussed |
| Abdullah Y, Schuman J, Shabsigh R | 2021 | Conceptual | Machine Learning | Screening,Diagnostic,Clinical monitoring,Treatment | Autonomy (including consent),Justice (equity or fairness),Bias,Accountability (ethically responsible for results),Privacy/Confidentiality,Transparency | Legal liability,Patient acceptability,Clinician acceptability,Reliability/accuracy |
| Melissa McCradden, Katrina Hui, Daniel Z Buchman | 2022 | Conceptual | AI Generally (no specific method used) | Screening,Diagnostic,Treatment | Autonomy (including consent),Justice (equity or fairness),Bias,Accountability (ethically responsible for results),Transparency | Legal liability,Patient acceptability,Barriers to implementation/adoption within the clinical context,Reliability/accuracy |
| Melissa McCradden, Katrina Hui, Daniel Z Buchman | 2022 | Conceptual | AI Generally (no specific method used) | Screening,Diagnostic,Clinical monitoring,Treatment | Autonomy (including consent),Justice (equity or fairness),Bias,Accountability (ethically responsible for results),Transparency | Legal liability,Patient acceptability,Barriers to implementation/adoption within the clinical context,Reliability/accuracy |
| Francis McKay, Bethany J Williams, Graham Prestwich, et al. | 2022 | Conceptual | Other | Diagnostic,Other/Non-specific | Autonomy (including consent),Justice (equity or fairness),Bias,Privacy/Confidentiality | Patient acceptability |
| Zhu, J; Shi, K; Yang, C | 2021 | Empirical | Other | Clinical monitoring | Autonomy (including consent),Justice (equity or fairness),Privacy/Confidentiality | Not discussed |
| Tayo Obafemi-Ajayi, Andy Perkins, Bindu Nanduri, et al. | 2021 | Conceptual | AI Generally (no specific method used) | Diagnostic,Treatment,Other/Non-specific | Justice (equity or fairness),Bias,Transparency | Barriers to implementation/adoption within the clinical context,Reliability/accuracy |
| Yoon,C; Torrance,R; Scheinerman, N | 2020 | Conceptual | Machine Learning | Diagnostic | Justice (equity or fairness),Bias,Privacy/Confidentiality,Transparency | Not discussed |
| Petersen, E Potdevin,Y Mohammadi, E , et al | 2022 | Empirical | AI Generally (no specific method used) | Screening,Diagnostic,Treatment | Autonomy (including consent),Justice (equity or fairness),Bias,Benefits,Privacy/Confidentiality,Transparency | Legal liability,Barriers to implementation/adoption within the clinical context,Reliability/accuracy |
| Ott, T Dabrock,P | 2022 | Conceptual | AI Generally (no specific method used) | Diagnostic,Treatment | Justice (equity or fairness),Bias,Benefits,Transparency | Patient acceptability,Barriers to implementation/adoption within the clinical context,Reliability/accuracy |
| Palmer,A Schwan,D | 2021 | Empirical | AI Generally (no specific method used) | Screening,Diagnostic,Treatment | Bias,Privacy/Confidentiality | Barriers to implementation/adoption within the clinical context |
| Ott, M | 2022 | Conceptual | Machine Learning | Diagnostic,Treatment | Autonomy (including consent),Justice (equity or fairness),Bias | Patient acceptability,Reliability/accuracy |
| Smallman, M. | 2022 | Conceptual | AI Generally (no specific method used) | Clinical monitoring | Justice (equity or fairness),Bias,Accountability (ethically responsible for results),Privacy/Confidentiality,Transparency | Patient acceptability,Reliability/accuracy |
| Sand, M., Duran, J.M., Jongsma, K.R. | 2022 | Conceptual | AI Generally (no specific method used) | Screening,Diagnostic,Clinical monitoring,Treatment | Accountability (ethically responsible for results),Disclosure of results/Return of results to patients,Other | Clinician acceptability,Barriers to implementation/adoption within the clinical context,Reliability/accuracy |
| Yang, L; Ene, I; Belaghi, R | 2021 | Empirical | AI Generally (no specific method used) | Diagnostic | Privacy/Confidentiality,Disclosure of results/Return of results to patients | Patient acceptability,Barriers to implementation/adoption within the clinical context |
| Michael Miller Jr | 2022 | Conceptual | AI Generally (no specific method used) | Screening,Diagnostic,Other/Non-specific | Bias,Privacy/Confidentiality,Other | Barriers to implementation/adoption within the clinical context |
| Rogers, W.A., Draper, H., Carter, S.M. | 2022 | Conceptual | AI Generally (no specific method used) | Diagnostic,Clinical monitoring | Privacy/Confidentiality,Transparency,Other | Patient acceptability,Clinician acceptability,Barriers to implementation/adoption within the clinical context,Reliability/accuracy |
| Milad Mirababaie, Lennart Hofeditz, Nicholad R. J. Frick, et al. | 2021 | Empirical | AI Generally (no specific method used) | Diagnostic,Clinical monitoring,Treatment | Autonomy (including consent),Justice (equity or fairness),Bias,Benefits,Accountability (ethically responsible for results),Privacy/Confidentiality,Transparency | Patient acceptability,Clinician acceptability,Barriers to implementation/adoption within the clinical context,Reliability/accuracy |
| Quinn, T.P., Jacobs, S., Senadeera, M., Le, V., Coghlan, S. | 2022 | Conceptual | Machine Learning | Other/Non-specific | Autonomy (including consent),Bias,Benefits,Accountability (ethically responsible for results),Transparency | Reliability/accuracy |
| Spector-Baagdady, K; Rahimzadeh, V; Jaffe, K; Moreno, J | 2022 | Conceptual | Machine Learning | Other/Non-specific | Autonomy (including consent),Justice (equity or fairness),Bias,Privacy/Confidentiality | Legal liability,Reliability/accuracy |
| Pham, Q; Gamble, A; Hearn, J | 2021 | Empirical | AI Generally (no specific method used) | Clinical monitoring | Bias | Reliability/accuracy |
| Chiang, Sharon; Picard, Rosalind; Chiong Winston; et al | 2021 | Conceptual | AI Generally (no specific method used) | Diagnostic,Treatment,Other/Non-specific | Autonomy (including consent),Justice (equity or fairness),Bias,Transparency,Other | Reliability/accuracy |
| Dankwa-Mullan, Irene; Scheufele, Elisabeth; Matheny, Michael; et al | 2021 | Conceptual | AI Generally (no specific method used) | Clinical monitoring,Treatment,Other/Non-specific | Justice (equity or fairness),Bias,Accountability (ethically responsible for results),Transparency,Other | Barriers to implementation/adoption within the clinical context |
| Kulikowski CA. | 2022 | Empirical | AI Generally (no specific method used) | Diagnostic | Autonomy (including consent),Justice (equity or fairness),Accountability (ethically responsible for results) | Not discussed |
| Nevanpera, M; Rajamaki, J; Helin, J | 2021 | Conceptual | AI Generally (no specific method used) | Clinical monitoring,Other/Non-specific | Justice (equity or fairness),Bias,Accountability (ethically responsible for results),Privacy/Confidentiality,Transparency | Not discussed |
| Boer, B Kudina,O | 2022 | Conceptual | Machine Learning | Diagnostic | Bias,Accountability (ethically responsible for results) | Legal liability,Patient acceptability,Clinician acceptability |
| Heyen, N.B. & Salloch, S. | 2021 | Conceptual | Machine Learning | Screening,Diagnostic,Clinical monitoring | Autonomy (including consent),Justice (equity or fairness),Bias,Benefits,Transparency | Patient acceptability,Clinician acceptability,Barriers to implementation/adoption within the clinical context |
| Murphy,K; Ruggiero, E; Upshur,R | 2021 | Empirical | AI Generally (no specific method used) | Diagnostic,Clinical monitoring,Treatment | Bias,Accountability (ethically responsible for results),Privacy/Confidentiality | Barriers to implementation/adoption within the clinical context |
| Lang BH. | 2022 | Conceptual | Other | Diagnostic | Accountability (ethically responsible for results),Transparency | Not discussed |
| Panfilis,L Peruselli,C Tanzi,S Botrungo,C | 2021 | Empirical | Machine Learning | Diagnostic,Clinical monitoring,Treatment | Bias,Privacy/Confidentiality,Disclosure of results/Return of results to patients | Legal liability,Clinician acceptability,Barriers to implementation/adoption within the clinical context,Reliability/accuracy |
| Duran,J Jongsma,K | 2021 | Conceptual | Machine Learning | Treatment | Autonomy (including consent),Bias,Accountability (ethically responsible for results) | Patient acceptability,Barriers to implementation/adoption within the clinical context,Reliability/accuracy |
| Gunderson, T., B?r?e, K. | 2022 | Conceptual | AI Generally (no specific method used) | Other/Non-specific | Bias,Accountability (ethically responsible for results),Transparency | Clinician acceptability,Reliability/accuracy |
| Felder,R | 2021 | Conceptual | Machine Learning | Diagnostic,Clinical monitoring,Treatment | Autonomy (including consent),Benefits,Accountability (ethically responsible for results),Transparency | Legal liability,Barriers to implementation/adoption within the clinical context,Reliability/accuracy |
| Lee, SS. | 2022 | Conceptual | AI Generally (no specific method used) | Other/Non-specific | Other | Not discussed |
| Lin, S. | 2022 | Conceptual | AI Generally (no specific method used) | Diagnostic,Other/Non-specific | Justice (equity or fairness),Bias | Reliability/accuracy |
| Ti, L., Ho, A., & Knight, R. | 2021 | Conceptual | AI Generally (no specific method used) | Screening,Diagnostic | Autonomy (including consent),Justice (equity or fairness),Bias,Transparency,Disclosure of results/Return of results to patients | Patient acceptability,Clinician acceptability,Reliability/accuracy |
| Sunarti, S.A., Rahmana, F.F., Muhammad, N.A., Muhammad, R., Kresna, F., Rusni, M. | 2020 | Conceptual | AI Generally (no specific method used) | Other/Non-specific | Autonomy (including consent),Justice (equity or fairness),Bias,Benefits,Accountability (ethically responsible for results),Transparency | Patient acceptability,Clinician acceptability,Barriers to implementation/adoption within the clinical context,Reliability/accuracy |
| G.T., K.G. | 2022 | Conceptual | Machine Learning | Diagnostic,Other/Non-specific | Justice (equity or fairness),Bias,Privacy/Confidentiality | Reliability/accuracy |
| Giovanola B, Tiribelli S. | 2022 | Conceptual | Machine Learning | Screening,Diagnostic,Other/Non-specific | Justice (equity or fairness),Bias,Transparency | Reliability/accuracy |
| Fleming, M | 2021 | Empirical | Machine Learning | Screening | Autonomy (including consent),Justice (equity or fairness),Privacy/Confidentiality | Clinician acceptability,Barriers to implementation/adoption within the clinical context,Reliability/accuracy |
| Hatherley, J., Sparrow, R., Howard, M. | 2022 | Conceptual | AI Generally (no specific method used) | Other/Non-specific | Benefits | Reliability/accuracy |
| Saheb,T , Saheb, T , Carpenter, D | 2021 | Empirical | AI Generally (no specific method used) | Diagnostic,Clinical monitoring,Treatment | Autonomy (including consent),Justice (equity or fairness),Bias | Legal liability,Patient acceptability,Barriers to implementation/adoption within the clinical context,Reliability/accuracy |
| Char, D., Abramoff, M., Feudtner, C. | 2020 | Empirical | Machine Learning | Screening,Diagnostic,Treatment,Other/Non-specific | Autonomy (including consent),Justice (equity or fairness),Bias,Benefits,Accountability (ethically responsible for results),Privacy/Confidentiality,Transparency | Reliability/accuracy |
| de Miguel Beriain, I. | 2020 | Conceptual | AI Generally (no specific method used) | Treatment | Autonomy (including consent) | Not discussed |
| Holohan, M. & Fiske, A. | 2021 | Conceptual | AI Generally (no specific method used) | Clinical monitoring,Treatment | Benefits,Transparency,Disclosure of results/Return of results to patients | Patient acceptability,Clinician acceptability,Barriers to implementation/adoption within the clinical context |
| Barron, D.S. | 2021 | Conceptual | Machine Learning | Screening,Diagnostic,Treatment | Justice (equity or fairness),Benefits,Accountability (ethically responsible for results) | Patient acceptability,Clinician acceptability,Reliability/accuracy |
| Rubeis, G. | 2020 | Conceptual | AI Generally (no specific method used) | Screening,Diagnostic,Clinical monitoring,Treatment | Autonomy (including consent),Justice (equity or fairness),Bias,Benefits,Transparency | Patient acceptability,Clinician acceptability,Barriers to implementation/adoption within the clinical context |
| Ferrario A. | 2022 | Conceptual | AI Generally (no specific method used) | Other/Non-specific | Transparency,Other | Reliability/accuracy |
| Ferryman K. | 2022 | Conceptual | AI Generally (no specific method used) | Other/Non-specific | Justice (equity or fairness),Privacy/Confidentiality,Other | Barriers to implementation/adoption within the clinical context,Reliability/accuracy |
| Findley J, Woods A, Robertson C, Slepian M. | 2020 | Conceptual | AI Generally (no specific method used) | Diagnostic,Treatment,Other/Non-specific | Autonomy (including consent),Benefits,Other | Patient acceptability |
| Fiske A, Tigard D, M?ller R, Haddadin S, Buyx A, McLennan S. | 2020 | Conceptual | AI Generally (no specific method used) | Other/Non-specific | Autonomy (including consent),Benefits,Privacy/Confidentiality,Other | Barriers to implementation/adoption within the clinical context |
| Gerhards H, Weber K, Bittner U, Fangerau H. | 2020 | Conceptual | AI Generally (no specific method used) | Other/Non-specific | Justice (equity or fairness),Bias,Other | Barriers to implementation/adoption within the clinical context,Reliability/accuracy |
| Grote T, Berens P. | 2020 | Conceptual | AI Generally (no specific method used) | Diagnostic,Other/Non-specific | Autonomy (including consent),Justice (equity or fairness),Benefits,Accountability (ethically responsible for results),Transparency | Legal liability,Reliability/accuracy |
| Braun M, Hummel P, Beck S, Dabrock P. | 2020 | Conceptual | AI Generally (no specific method used) | Other/Non-specific | Bias,Benefits,Privacy/Confidentiality,Transparency | Legal liability,Barriers to implementation/adoption within the clinical context,Reliability/accuracy |
| Ienca M, Ignatiadis K. | 2020 | Conceptual | Machine Learning | Diagnostic | Justice (equity or fairness),Bias,Accountability (ethically responsible for results),Privacy/Confidentiality | Not discussed |
| Canales C, Lee C, Cannesson M. | 2020 | Conceptual | Machine Learning | Diagnostic,Treatment | Justice (equity or fairness),Bias,Privacy/Confidentiality,Transparency | Legal liability,Reliability/accuracy |
| Karnik NS, Afshar M, Churpek MM, Nunez-Smith M. | 2020 | Conceptual | Machine Learning | Diagnostic | Justice (equity or fairness),Bias | Not discussed |
| Keskinbora K, G?ven F. | 2020 | Conceptual | AI Generally (no specific method used) | Diagnostic | Bias | Not discussed |
| Fenech M.E., Buston, O. | 2020 | Conceptual | AI Generally (no specific method used) | Other/Non-specific | Autonomy (including consent),Bias,Benefits,Accountability (ethically responsible for results),Privacy/Confidentiality,Transparency,Disclosure of results/Return of results to patients | Legal liability,Patient acceptability,Clinician acceptability,Reliability/accuracy |
| Ferrario A, Loi M, Vigan? E. | 2020 | Conceptual | AI Generally (no specific method used) | Other/Non-specific | Other | Not discussed |
| Char D, Abr?moff M, Feudtner C. | 2020 | Conceptual | Machine Learning | Other/Non-specific | Justice (equity or fairness),Bias,Accountability (ethically responsible for results) | Barriers to implementation/adoption within the clinical context |
| Kluge EW. | 2020 | Conceptual | AI Generally (no specific method used) | Screening,Diagnostic | Autonomy (including consent),Bias,Accountability (ethically responsible for results),Privacy/Confidentiality | Not discussed |
| Kraft SA. | 2020 | Conceptual | Machine Learning | Other/Non-specific | Other | Not discussed |
| Isbanner S, O'Shaughnessy P, Steel D, Wilcock S, Carter S. | 2022 | Empirical | AI Generally (no specific method used) | Screening,Diagnostic,Treatment,Other/Non-specific | Justice (equity or fairness),Benefits,Accountability (ethically responsible for results),Privacy/Confidentiality,Transparency,Other | Reliability/accuracy |
| Jobson D, Mar V, Freckelton I. | 2022 | Conceptual | AI Generally (no specific method used) | Screening,Diagnostic,Other/Non-specific | Autonomy (including consent),Benefits,Accountability (ethically responsible for results),Privacy/Confidentiality,Transparency | Legal liability,Reliability/accuracy |
| Habli I, Lawton T, Porter Z. | 2020 | Conceptual | AI Generally (no specific method used) | Treatment,Other/Non-specific | Bias,Benefits,Accountability (ethically responsible for results) | Reliability/accuracy |
| Hardt M, Chin MH. | 2020 | Conceptual | AI Generally (no specific method used) | Other/Non-specific | Justice (equity or fairness),Bias,Transparency,Other | Not discussed |
| Hatherley JJ. | 2020 | Conceptual | AI Generally (no specific method used) | Other/Non-specific | Accountability (ethically responsible for results) | Patient acceptability,Reliability/accuracy |
| Heinrichs B, Eickhoff SB. | 2020 | Conceptual | AI Generally (no specific method used) | Other/Non-specific | Accountability (ethically responsible for results),Transparency | Reliability/accuracy |
| Ho A. | 2020 | Conceptual | AI Generally (no specific method used) | Other/Non-specific | Autonomy (including consent),Justice (equity or fairness),Privacy/Confidentiality,Other | Patient acceptability,Barriers to implementation/adoption within the clinical context |
| Biller-Andorno N, Ferrario A, Joebges S, et al. | 2020 | Empirical | Machine Learning | Other/Non-specific | Justice (equity or fairness),Bias,Benefits,Accountability (ethically responsible for results),Transparency | Legal liability,Patient acceptability,Clinician acceptability,Barriers to implementation/adoption within the clinical context,Reliability/accuracy |
| Abramoff, M., Cunningham, B., Patel, B. | 2022 | Conceptual | AI Generally (no specific method used) | Diagnostic | Autonomy (including consent),Justice (equity or fairness),Bias,Benefits,Accountability (ethically responsible for results),Transparency | Legal liability,Reliability/accuracy |
| Arnold MH. | 2021 | Conceptual | AI Generally (no specific method used) | Screening,Clinical monitoring,Treatment,Other/Non-specific | Autonomy (including consent),Justice (equity or fairness),Bias,Benefits,Accountability (ethically responsible for results),Privacy/Confidentiality,Transparency | Legal liability,Barriers to implementation/adoption within the clinical context,Reliability/accuracy |
| Chiang S, Picard RW, Chiong W, Moss R, Worrell GA, Rao VR, Goldenholz DM. | 2021 | Conceptual | AI Generally (no specific method used) | Diagnostic,Treatment | Justice (equity or fairness),Bias,Benefits,Accountability (ethically responsible for results),Privacy/Confidentiality,Transparency | Legal liability,Reliability/accuracy |
| Starke G, De Clercq E, Borgwardt S, Elger BS. | 2021 | Conceptual | AI Generally (no specific method used) | Diagnostic,Treatment,Other/Non-specific | Autonomy (including consent),Justice (equity or fairness),Bias,Benefits,Privacy/Confidentiality,Transparency | Barriers to implementation/adoption within the clinical context |
| Saheb T, Saheb T, Carpenter DO. | 2021 | Empirical | AI Generally (no specific method used) | Screening,Diagnostic,Treatment,Other/Non-specific | Autonomy (including consent),Justice (equity or fairness),Bias,Benefits,Accountability (ethically responsible for results),Privacy/Confidentiality,Transparency | Legal liability,Patient acceptability,Reliability/accuracy |
| Starke G, De Clercq E, Elger BS. | 2021 | Conceptual | Machine Learning | Diagnostic | Bias | Not discussed |
| Stewart C, Wong SKY, Sung JJY. | 2021 | Conceptual | AI Generally (no specific method used) | Diagnostic,Treatment | Autonomy (including consent),Justice (equity or fairness),Benefits,Accountability (ethically responsible for results),Privacy/Confidentiality | Legal liability,Patient acceptability |
| Uusitalo S, Tuominen J, Arstila V. | 2021 | Conceptual | AI Generally (no specific method used) | Diagnostic,Treatment | Accountability (ethically responsible for results) | Not discussed |
| Wadden JJ. | 2021 | Conceptual | AI Generally (no specific method used) | Diagnostic | Transparency | Not discussed |
| Ahmad, OF; Stoyanov, D; Lovat, LB | 2020 | Conceptual | AI Generally (no specific method used) | Diagnostic,Other/Non-specific | Autonomy (including consent),Bias,Accountability (ethically responsible for results),Privacy/Confidentiality,Transparency | Not discussed |
| Arambula AM, Bur AM. | 2020 | Conceptual | AI Generally (no specific method used) | Diagnostic | Autonomy (including consent),Justice (equity or fairness),Bias,Privacy/Confidentiality | Not discussed |
| Palmer A. Schwan D, | 2021 | Conceptual | AI Generally (no specific method used) | Clinical monitoring,Treatment | Justice (equity or fairness),Benefits,Disclosure of results/Return of results to patients | Patient acceptability,Clinician acceptability,Barriers to implementation/adoption within the clinical context |
| Yirmibesoglu Erkal W, Akpinar A, Sukru Erkal, H. | 2021 | Conceptual | AI Generally (no specific method used) | Treatment,Other/Non-specific | Autonomy (including consent),Justice (equity or fairness),Bias,Accountability (ethically responsible for results),Other | Reliability/accuracy |
| Ursin F, Timmermann C, Steger F | 2021 | Empirical | AI Generally (no specific method used) | Other/Non-specific | Transparency | Reliability/accuracy |
| de Boer B, Kudina O. | 2021 | Conceptual | Machine Learning | Diagnostic | Bias,Accountability (ethically responsible for results),Other | Legal liability |
| Abramoff M, Tobey D, Char D | 2020 | Empirical | AI Generally (no specific method used) | Screening,Diagnostic | Autonomy (including consent),Bias,Benefits,Accountability (ethically responsible for results),Privacy/Confidentiality,Transparency | Legal liability,Clinician acceptability,Reliability/accuracy |
| Dur?n JM, Jongsma KR. | 2021 | Conceptual | AI Generally (no specific method used) | Screening,Diagnostic,Treatment | Autonomy (including consent),Accountability (ethically responsible for results),Transparency | Not discussed |
| Felder RM. | 2021 | Conceptual | AI Generally (no specific method used) | Diagnostic | Accountability (ethically responsible for results) | Not discussed |
| Starke G, Ienca, M | 2022 | Conceptual | AI Generally (no specific method used) | Other/Non-specific | Transparency | Barriers to implementation/adoption within the clinical context |
| Mirbabaie, M., Hofeditz, L., Frick, N.R.J., Stieglitz, S. | 2022 | Conceptual | AI Generally (no specific method used) | Diagnostic,Clinical monitoring,Treatment | Autonomy (including consent),Justice (equity or fairness),Benefits,Privacy/Confidentiality | Legal liability,Patient acceptability,Clinician acceptability,Reliability/accuracy |
| Chauhan, C. & Gullapalli, R.R. | 2021 | Conceptual | AI Generally (no specific method used) | Diagnostic,Clinical monitoring | Justice (equity or fairness),Bias,Accountability (ethically responsible for results),Transparency,Other | Legal liability,Barriers to implementation/adoption within the clinical context |
| Chen, I., Pierson, E., Rose, S., Joshi, S., Ferryman, K., & Ghassemi, M. | 2021 | Conceptual | Machine Learning | Screening,Diagnostic,Clinical monitoring,Treatment | Justice (equity or fairness),Accountability (ethically responsible for results),Transparency | Barriers to implementation/adoption within the clinical context,Reliability/accuracy |
| Starke G, Poppe C. | 2022 | Conceptual | AI Generally (no specific method used) | Other/Non-specific | Transparency | Not discussed |
| Kenny L, Nevin M, Fitzpatrick K. | 2021 | Empirical | Machine Learning | Other/Non-specific | Autonomy (including consent),Bias,Accountability (ethically responsible for results),Privacy/Confidentiality,Transparency | Legal liability,Reliability/accuracy |
| Mirbabais, M., Hofeditz, L., Frick, N., R., Stieglitz, S., | 2022 | Empirical | AI Generally (no specific method used) | Treatment | Autonomy (including consent),Justice (equity or fairness),Bias,Benefits,Accountability (ethically responsible for results),Privacy/Confidentiality,Transparency,Other | Legal liability |
| Chen, I., Y., Pierson, E., Rose, S., Joshi, S., Ferryman, K., Ghassemi, M. | 2021 | Conceptual | Machine Learning | Other/Non-specific | Justice (equity or fairness),Other | Reliability/accuracy |
| Miller M. | 2022 | Conceptual | Machine Learning | Other/Non-specific | Accountability (ethically responsible for results),Privacy/Confidentiality,Transparency,Other | Reliability/accuracy |
| obafemi-Ajayi, T., Perkins, A., Nanduri, B., Wunsch, D. C., Foster, J., A., Peckham, J. | 2022 | Conceptual | Machine Learning | Screening,Diagnostic,Treatment | Autonomy (including consent),Justice (equity or fairness),Bias,Accountability (ethically responsible for results),Transparency,Other | Reliability/accuracy |
| Melanie Smallman | 2022 | Conceptual | AI Generally (no specific method used) | Other/Non-specific | Justice (equity or fairness),Benefits,Accountability (ethically responsible for results),Privacy/Confidentiality | Not discussed |
| Martinez-Martin N, Luo Z, Kaushal A, et al. | 2020 | Conceptual | AI Generally (no specific method used) | Other/Non-specific | Autonomy (including consent),Bias,Privacy/Confidentiality,Transparency | Legal liability,Patient acceptability,Barriers to implementation/adoption within the clinical context,Reliability/accuracy |
| Mouchabac S, Adrien V, Falala-S?chet C, Bonnot O, Maatoug R, Millet B, Peretti CS, Bourla A, Ferreri F. | 2021 | Conceptual | Machine Learning | Diagnostic,Other/Non-specific | Autonomy (including consent),Justice (equity or fairness),Benefits,Privacy/Confidentiality | Reliability/accuracy |
| Asan O, Bayrak AE, Choudhury A. | 2020 | Conceptual | AI Generally (no specific method used) | Other/Non-specific | Justice (equity or fairness),Transparency | Clinician acceptability,Reliability/accuracy |
| Blobel B, Ruotsalainen P, Brochhausen M, Oemig F, Uribe GA. | 2020 | Conceptual | Other | Other/Non-specific | Accountability (ethically responsible for results),Privacy/Confidentiality,Transparency,Other | Not discussed |
| Brady AP, Neri E. | 2020 | Conceptual | AI Generally (no specific method used) | Other/Non-specific | Autonomy (including consent),Justice (equity or fairness),Bias,Privacy/Confidentiality,Transparency,Other | Legal liability,Patient acceptability |
| Yang L, Ene IC, Arabi Belaghi R, Koff D, Stein N, Santaguida PL. | 2022 | Empirical | AI Generally (no specific method used) | Diagnostic | Autonomy (including consent),Accountability (ethically responsible for results),Privacy/Confidentiality | Not discussed |
| De Panfilis L, Peruselli C, Tanzi S, Botrugno C. | 2021 | Empirical | AI Generally (no specific method used) | Screening,Diagnostic | Autonomy (including consent),Bias,Benefits,Accountability (ethically responsible for results),Privacy/Confidentiality,Transparency | Not discussed |
| Fleming MN. | 2021 | Conceptual | Machine Learning | Diagnostic | Autonomy (including consent),Privacy/Confidentiality | Not discussed |
| Ott T, Dabrock P. | 2022 | Conceptual | AI Generally (no specific method used) | Other/Non-specific | Transparency | Not discussed |
| Pirni A, Balistreri M, Capasso M, Umbrello S, Merenda F | 2021 | Conceptual | AI Generally (no specific method used) | Clinical monitoring,Treatment | Autonomy (including consent),Justice (equity or fairness),Disclosure of results/Return of results to patients | Patient acceptability,Reliability/accuracy |
| Neri E, Coppola F, Miele V, Bibbolino B, Grassi R | 2020 | Conceptual | AI Generally (no specific method used) | Screening,Diagnostic,Clinical monitoring,Treatment | Autonomy (including consent),Bias,Accountability (ethically responsible for results),Privacy/Confidentiality,Transparency | Legal liability,Clinician acceptability,Barriers to implementation/adoption within the clinical context,Reliability/accuracy |
| Morley J, Machado C, Burr C, Cowls J, Joshi I, Taddeo M, Floridi L | 2020 | Conceptual | AI Generally (no specific method used) | Screening,Diagnostic,Clinical monitoring,Treatment | Autonomy (including consent),Justice (equity or fairness),Bias,Benefits,Accountability (ethically responsible for results),Privacy/Confidentiality,Transparency | Legal liability,Barriers to implementation/adoption within the clinical context,Reliability/accuracy |
| McFarlane J, Illes J. | 2020 | Empirical | Machine Learning | Diagnostic,Treatment | Justice (equity or fairness),Bias,Privacy/Confidentiality | Barriers to implementation/adoption within the clinical context,Reliability/accuracy |
| Kerasidou CX, Kerasidou A, Buscher M, Wilkinson S. | 2022 | Conceptual | AI Generally (no specific method used) | Diagnostic | Autonomy (including consent),Benefits,Accountability (ethically responsible for results),Privacy/Confidentiality,Transparency | Legal liability,Barriers to implementation/adoption within the clinical context |
| Hallows R, Glazier L, Katz MS, Aznar M, Williams M. | 2022 | Empirical | AI Generally (no specific method used) | Other/Non-specific | Autonomy (including consent),Justice (equity or fairness),Bias,Benefits,Accountability (ethically responsible for results),Transparency | Clinician acceptability,Reliability/accuracy |
| Dave P, Nambudiri V, Grant-Kels JM. | 2022 | Empirical | AI Generally (no specific method used) | Screening,Diagnostic | Autonomy (including consent),Justice (equity or fairness),Bias,Benefits,Transparency | Clinician acceptability,Barriers to implementation/adoption within the clinical context,Reliability/accuracy |
| McLennan S, Lee MM, Fiske A, Celi LA. | 2020 | Empirical | Machine Learning | Clinical monitoring,Treatment | Bias,Accountability (ethically responsible for results),Transparency | Clinician acceptability,Barriers to implementation/adoption within the clinical context,Reliability/accuracy |
| Jotterand, F; Bosco, C | 2021 | Conceptual | AI Generally (no specific method used) | Other/Non-specific | Bias,Benefits,Accountability (ethically responsible for results),Transparency | Patient acceptability,Barriers to implementation/adoption within the clinical context,Reliability/accuracy |
| Kasperbauer, T.J. | 2020 | Conceptual | AI Generally (no specific method used) | Screening,Diagnostic,Clinical monitoring,Treatment,Other/Non-specific | Bias,Accountability (ethically responsible for results),Transparency,Other | Clinician acceptability,Barriers to implementation/adoption within the clinical context,Reliability/accuracy |
| Caffery, LJ; Janda, M; Miller, R; Abbott, LM; Arnold, C; Caccetta, T; Guitera, P; Shumack, S; Fernandez-Penas, P; Mar, V; Soyer, HP | 2022 | Empirical | Machine Learning | Screening,Diagnostic,Clinical monitoring,Treatment | Autonomy (including consent),Justice (equity or fairness),Bias,Accountability (ethically responsible for results),Privacy/Confidentiality,Transparency,Disclosure of results/Return of results to patients | Legal liability |
| Mazurowski, M | 2020 | Conceptual | Other | Screening,Diagnostic,Clinical monitoring,Treatment | Autonomy (including consent),Justice (equity or fairness),Bias,Benefits | Clinician acceptability,Barriers to implementation/adoption within the clinical context |
| Villongco C, Khan F. | 2020 | Conceptual | AI Generally (no specific method used) | Screening,Diagnostic | Justice (equity or fairness),Bias,Benefits | Patient acceptability |
| McCradden, M; Anderson, J; Zlotnik Shaul, R | 2020 | Empirical | Machine Learning | Clinical monitoring | Justice (equity or fairness),Bias,Accountability (ethically responsible for results) | Barriers to implementation/adoption within the clinical context,Reliability/accuracy |
| Shaw JA, Sethi N, Block BL. | 2021 | Conceptual | AI Generally (no specific method used) | Other/Non-specific | Autonomy (including consent),Justice (equity or fairness),Bias,Accountability (ethically responsible for results) | Patient acceptability |
| Pot M, Kieusseyan N, Prainsack B. | 2021 | Conceptual | Machine Learning | Diagnostic | Justice (equity or fairness),Bias,Accountability (ethically responsible for results),Transparency | Not discussed |
| Liu, T.Y.& Bressler, Neil | 2020 | Conceptual | AI Generally (no specific method used) | Screening,Diagnostic | Bias,Accountability (ethically responsible for results),Privacy/Confidentiality,Other | Legal liability,Clinician acceptability,Barriers to implementation/adoption within the clinical context,Reliability/accuracy |
| Sudip Bhattacharya, Md Mahbub Hossain1, Ruchi Juyal, Neha Sharma, Keerti Bhusan Pradhan2, Amarjeet Singh | 2021 | Conceptual | AI Generally (no specific method used) | Other/Non-specific | Bias,Benefits,Accountability (ethically responsible for results) | Legal liability,Reliability/accuracy |
| Chu CH, Leslie K, Shi J, Nyrup R, Bianchi A, Khan SS, Rahimi SA, Lyn A, Grenier A. | 2022 | Empirical | Machine Learning | Other/Non-specific | Bias | Legal liability |
| Anto Cartolovni, Ana Tomicic, Elvira Lazic Mosler | 2022 | Empirical | AI Generally (no specific method used) | Diagnostic,Clinical monitoring,Treatment | Accountability (ethically responsible for results),Transparency | Legal liability,Clinician acceptability,Barriers to implementation/adoption within the clinical context,Reliability/accuracy |
| McCradden MD, Joshi S, Mazwi M, Anderson JA. | 2020 | Conceptual | AI Generally (no specific method used) | Diagnostic,Clinical monitoring | Justice (equity or fairness),Bias,Transparency | Reliability/accuracy |
| M?llmann NR, Mirbabaie M, Stieglitz S. | 2021 | Empirical | Machine Learning | Other/Non-specific | Autonomy (including consent),Justice (equity or fairness),Benefits | Legal liability |
| Melissa D. McCradden, Ami Baba, Ashirbani Saha, et al. | 2020 | Empirical | AI Generally (no specific method used) | Treatment,Other/Non-specific | Autonomy (including consent),Privacy/Confidentiality,Transparency | Patient acceptability,Clinician acceptability |
| Antoniadi, AM; Du, YH; Guendouz, Y; Wei, L; Mazo, C; Becker, BA; Mooney, C | 2021 | Conceptual | Machine Learning | Screening,Diagnostic,Clinical monitoring,Treatment | Justice (equity or fairness),Benefits,Transparency,Disclosure of results/Return of results to patients | Patient acceptability,Clinician acceptability,Barriers to implementation/adoption within the clinical context |
| Fletcher RR, Nakeshimana A, Olubeko O. | 2021 | Conceptual | AI Generally (no specific method used) | Screening,Diagnostic,Clinical monitoring,Treatment | Justice (equity or fairness),Bias,Other | Legal liability,Patient acceptability,Clinician acceptability,Barriers to implementation/adoption within the clinical context |
| Ho CW, Caals K. | 2021 | Conceptual | AI Generally (no specific method used) | Screening,Diagnostic,Clinical monitoring,Treatment | Benefits,Transparency,Other | Barriers to implementation/adoption within the clinical context |
| Overton DJ. | 2020 | Conceptual | AI Generally (no specific method used) | Screening,Clinical monitoring,Other/Non-specific | Autonomy (including consent),Benefits,Privacy/Confidentiality,Transparency | Not discussed |
| M?rch CM, Gupta A, Mishara BL. | 2020 | Conceptual | AI Generally (no specific method used) | Screening,Clinical monitoring | Accountability (ethically responsible for results) | Not discussed |
| Nabi J. | 2020 | Conceptual | Machine Learning | Other/Non-specific | Bias,Transparency | Reliability/accuracy |
| Blumenthal-Barby, J. | 2022 | Conceptual | AI Generally (no specific method used) | Diagnostic,Treatment | Autonomy (including consent),Justice (equity or fairness),Bias,Privacy/Confidentiality,Transparency | Patient acceptability,Clinician acceptability |
| Crossnohere, N.; Elsaid, M.; Paskett, J. | 2022 | Empirical | AI Generally (no specific method used) | Diagnostic,Treatment,Other/Non-specific | Autonomy (including consent),Privacy/Confidentiality,Transparency | Reliability/accuracy,Not discussed |
| Skorburg, J | 2020 | Conceptual | Machine Learning | Diagnostic,Treatment | Bias | Not discussed |
| Stokes F, Palmer A. | 2020 | Conceptual | AI Generally (no specific method used) | Diagnostic | Privacy/Confidentiality | Barriers to implementation/adoption within the clinical context |
| Luxton DD. | 2022 | Conceptual | AI Generally (no specific method used) | Screening,Diagnostic,Clinical monitoring,Treatment | Benefits,Accountability (ethically responsible for results),Transparency,Disclosure of results/Return of results to patients | Patient acceptability,Clinician acceptability,Barriers to implementation/adoption within the clinical context |
| Pedersen M, Verspoor K, Jenkinson M, Law M, Abbott DF, Jackson GD. | 2020 | Conceptual | AI Generally (no specific method used) | Diagnostic | Justice (equity or fairness),Accountability (ethically responsible for results),Privacy/Confidentiality,Transparency,Disclosure of results/Return of results to patients | Legal liability,Patient acceptability,Clinician acceptability,Barriers to implementation/adoption within the clinical context |
| Reddy S, Allan S, Coghlan S, Cooper P. | 2020 | Conceptual | AI Generally (no specific method used) | Other/Non-specific | Justice (equity or fairness),Bias,Accountability (ethically responsible for results),Privacy/Confidentiality,Transparency,Disclosure of results/Return of results to patients | Barriers to implementation/adoption within the clinical context |
| McCradden M, Joshi S, Anderson J, et al. | 2020 | Conceptual | Machine Learning | Other/Non-specific | Justice (equity or fairness),Bias,Accountability (ethically responsible for results),Other | Legal liability,Reliability/accuracy |
| Straw, I. | 2021 | Empirical | AI Generally (no specific method used) | Screening | Autonomy (including consent),Justice (equity or fairness),Bias,Accountability (ethically responsible for results),Privacy/Confidentiality,Other | Legal liability |
| Ursin F, Timmermann C, Orzechowski M, & Steger F. | 2021 | Empirical | AI Generally (no specific method used) | Screening,Diagnostic | Autonomy (including consent),Justice (equity or fairness),Bias,Accountability (ethically responsible for results),Privacy/Confidentiality,Transparency | Clinician acceptability,Barriers to implementation/adoption within the clinical context |
| Zawati M, Lang N | 2020 | Conceptual | Machine Learning | Other/Non-specific | Accountability (ethically responsible for results),Transparency | Not discussed |
| Milton, C | 2021 | Conceptual | AI Generally (no specific method used) | Diagnostic,Clinical monitoring,Treatment | Autonomy (including consent),Benefits,Accountability (ethically responsible for results),Privacy/Confidentiality,Other | Clinician acceptability,Barriers to implementation/adoption within the clinical context,Reliability/accuracy |
| Rocheteau, E | 2022 | Conceptual | AI Generally (no specific method used) | Diagnostic,Clinical monitoring,Treatment,Other/Non-specific | Autonomy (including consent),Benefits,Accountability (ethically responsible for results),Privacy/Confidentiality | Legal liability,Clinician acceptability,Reliability/accuracy |
| Safdar, NM; Banja, JD; Meltzer, CC | 2020 | Conceptual | AI Generally (no specific method used) | Other/Non-specific | Justice (equity or fairness),Bias,Transparency | Legal liability,Clinician acceptability,Barriers to implementation/adoption within the clinical context |
| Crigger, E., Reinbold, K., Hanson, C., Kao, A., Blake, K., & Irons, M. | 2022 | Empirical | AI Generally (no specific method used) | Other/Non-specific | Justice (equity or fairness),Bias,Transparency | Reliability/accuracy |
| Prakash, S., Balaji, J.N., Joshi, A., Surapaneni, K.M. | 2022 | Conceptual | AI Generally (no specific method used) | Other/Non-specific | Autonomy (including consent),Justice (equity or fairness),Bias,Benefits,Accountability (ethically responsible for results),Privacy/Confidentiality,Transparency | Legal liability,Patient acceptability,Barriers to implementation/adoption within the clinical context,Reliability/accuracy |
| Chauhan, C., Gullapalli, R., R. | 2021 | Empirical | AI Generally (no specific method used) | Screening,Diagnostic,Clinical monitoring,Treatment | Autonomy (including consent),Justice (equity or fairness),Bias,Accountability (ethically responsible for results),Privacy/Confidentiality,Transparency,Other | Legal liability,Reliability/accuracy |
| Kostick-Quenet KM, Cohen IG, Gerke S, Lo B, Antaki J, Movahedi F, Njah H, Schoen L, Estep JE, Blumenthal-Barby JS. | 2022 | Conceptual | AI Generally (no specific method used) | Treatment,Other/Non-specific | Justice (equity or fairness),Bias | Legal liability,Reliability/accuracy |
| Kolansa K, Chabbert-Buffet N, Darai E, Antoina JA. | 2021 | Conceptual | AI Generally (no specific method used) | Diagnostic,Treatment,Other/Non-specific | Bias,Privacy/Confidentiality,Transparency | Clinician acceptability,Barriers to implementation/adoption within the clinical context |
| Lewis, A. | 2020 | Conceptual | AI Generally (no specific method used) | Clinical monitoring,Other/Non-specific | Justice (equity or fairness),Bias,Other | Reliability/accuracy |
| Svensson AM, Jotterand F. | 2022 | Conceptual | AI Generally (no specific method used) | Other/Non-specific | Autonomy (including consent),Justice (equity or fairness),Bias,Privacy/Confidentiality,Transparency,Disclosure of results/Return of results to patients | Reliability/accuracy |
| Grosek, S. | 2024 | Empirical | AI Generally (no specific method used) | Other/Non-specific | Autonomy (including consent),Justice (equity or fairness),Bias,Benefits,Accountability (ethically responsible for results),Privacy/Confidentiality,Transparency,Disclosure of results/Return of results to patients | Legal liability,Clinician acceptability,Barriers to implementation/adoption within the clinical context,Reliability/accuracy |
| B?lisle-Pipon, JC, Powell, M, Engilish R, et al. | 2024 | Empirical | AI Generally (no specific method used) | Other/Non-specific | Autonomy (including consent),Justice (equity or fairness),Bias,Benefits,Accountability (ethically responsible for results),Privacy/Confidentiality,Transparency | Legal liability,Reliability/accuracy |
| Gallagher A | 2024 | Conceptual | AI Generally (no specific method used) | Other/Non-specific | Justice (equity or fairness),Accountability (ethically responsible for results),Privacy/Confidentiality,Transparency | Not discussed |
| Giovanola B, Tiribelli S | 2023 | Conceptual | Machine Learning | Other/Non-specific | Justice (equity or fairness),Bias | Not discussed |
| Goirand M, Austin E, Clay-Williams R | 2023 | Empirical | AI Generally (no specific method used) | Diagnostic,Clinical monitoring,Treatment | Benefits,Privacy/Confidentiality,Transparency,Other | Not discussed |
| Gozum IEA, Flake CCD | 2024 | Conceptual | AI Generally (no specific method used) | Other/Non-specific | Justice (equity or fairness),Bias,Benefits,Transparency,Disclosure of results/Return of results to patients | Not discussed |
| Grzybowski, Andrezej | 2024 | Conceptual | AI Generally (no specific method used) | Other/Non-specific | Autonomy (including consent),Justice (equity or fairness),Bias,Accountability (ethically responsible for results),Privacy/Confidentiality,Transparency,Disclosure of results/Return of results to patients | Patient acceptability,Clinician acceptability,Barriers to implementation/adoption within the clinical context,Reliability/accuracy |
| Harishbhai Tilala, Mitul | 2024 | Conceptual | AI Generally (no specific method used) | Other/Non-specific | Autonomy (including consent),Justice (equity or fairness),Bias,Benefits,Accountability (ethically responsible for results),Privacy/Confidentiality,Transparency,Disclosure of results/Return of results to patients,Other | Legal liability,Patient acceptability,Clinician acceptability,Barriers to implementation/adoption within the clinical context,Reliability/accuracy |
| Heinrichs, J. | 2024 | Conceptual | AI Generally (no specific method used) | Other/Non-specific | Justice (equity or fairness),Bias,Accountability (ethically responsible for results) | Reliability/accuracy |
| Ford, E., Milne, R., & Curlewis, K. | 2023 | Conceptual | AI Generally (no specific method used) | Screening,Diagnostic,Clinical monitoring | Autonomy (including consent),Justice (equity or fairness),Benefits,Accountability (ethically responsible for results),Privacy/Confidentiality,Transparency | Patient acceptability,Clinician acceptability,Barriers to implementation/adoption within the clinical context,Reliability/accuracy |
| Freyer N., Grob D., & Lipprandt M. | 2024 | Empirical | Machine Learning | Other/Non-specific | Autonomy (including consent),Transparency,Other | Patient acceptability,Clinician acceptability,Reliability/accuracy |
| B?eliste-Pipon, JC | 2024 | Conceptual | Other | Other/Non-specific | Bias,Accountability (ethically responsible for results) | Barriers to implementation/adoption within the clinical context,Reliability/accuracy |
| Fritzsche M-C, Aky?z K, Cano Abad?a M, McLennan S, Marttinen P, Mayrhofer MT and Buyx AM | 2023 | Conceptual | Machine Learning | Diagnostic | Autonomy (including consent),Justice (equity or fairness),Bias,Privacy/Confidentiality | Patient acceptability,Clinician acceptability,Barriers to implementation/adoption within the clinical context |
| Hendricks-Sturrup R et al | 2023 | Empirical | AI Generally (no specific method used) | Other/Non-specific | Justice (equity or fairness),Bias,Benefits | Patient acceptability,Barriers to implementation/adoption within the clinical context |
| Elyoseph, Z., Shoval, D., Levkovich, I. | 2024 | Conceptual | Other | Other/Non-specific | Other | Not discussed |
| Yan, C. | 2024 | Conceptual | Other | Diagnostic,Other/Non-specific | Privacy/Confidentiality | Patient acceptability |
| Hesjedal MB et al | 2023 | Empirical | AI Generally (no specific method used) | Diagnostic | Benefits,Accountability (ethically responsible for results),Other | Patient acceptability,Clinician acceptability |
| Esmaeilzadeh, P. | 2024 | Conceptual | AI Generally (no specific method used) | Screening,Diagnostic,Clinical monitoring,Treatment | Justice (equity or fairness),Bias,Accountability (ethically responsible for results),Privacy/Confidentiality | Legal liability,Patient acceptability,Clinician acceptability,Barriers to implementation/adoption within the clinical context,Reliability/accuracy |
| Ho A, Bavli I, Mahal R, McKeown MJ | 2024 | Empirical | AI Generally (no specific method used) | Clinical monitoring | Autonomy (including consent),Benefits,Privacy/Confidentiality | Patient acceptability,Clinician acceptability,Barriers to implementation/adoption within the clinical context,Reliability/accuracy |
| Chustecki,, M. | 2024 | Empirical | AI Generally (no specific method used) | Diagnostic,Treatment,Other/Non-specific | Autonomy (including consent),Justice (equity or fairness),Bias,Accountability (ethically responsible for results),Privacy/Confidentiality,Transparency | Reliability/accuracy" |
| Hofweber T, Walker RL | 2024 | Conceptual | Machine Learning | Other/Non-specific | Autonomy (including consent),Justice (equity or fairness),Bias,Privacy/Confidentiality,Transparency,Other | Patient acceptability |
| Holm S | 2023 | Conceptual | AI Generally (no specific method used) | Other/Non-specific | Accountability (ethically responsible for results),Disclosure of results/Return of results to patients | Patient acceptability,Barriers to implementation/adoption within the clinical context |
| Fangerau, H. | 2024 | Conceptual | AI Generally (no specific method used) | Diagnostic,Clinical monitoring,Treatment,Other/Non-specific | Autonomy (including consent),Bias,Accountability (ethically responsible for results),Privacy/Confidentiality | Patient acceptability,Clinician acceptability,Barriers to implementation/adoption within the clinical context,Reliability/accuracy |
| Elendu, C. | 2023 | Empirical | AI Generally (no specific method used) | Other/Non-specific | Justice (equity or fairness),Bias,Accountability (ethically responsible for results),Privacy/Confidentiality,Transparency,Other | Legal liability |
| Federico, C. & Trotsyuk, A. | 2024 | Conceptual | AI Generally (no specific method used) | Screening,Other/Non-specific | Autonomy (including consent),Justice (equity or fairness),Bias,Privacy/Confidentiality | Legal liability |
| Ferryman, K.; Cesare, N.; Creary, M.; Nsoesie, E. | 2024 | Conceptual | AI Generally (no specific method used) | Other/Non-specific | Justice (equity or fairness),Bias | Not discussed |
| Funer F., Tinnemeyer S., Liedtke W., and Salloch S. | 2024 | Empirical | Machine Learning | Screening,Diagnostic,Clinical monitoring,Treatment | Accountability (ethically responsible for results),Other | Legal liability,Clinician acceptability,Barriers to implementation/adoption within the clinical context,Reliability/accuracy |
| Funer F., Liedtke W., Tinnemeyer S. | 2023 | Empirical | Machine Learning | Screening,Diagnostic,Clinical monitoring,Treatment | Accountability (ethically responsible for results),Other | Legal liability,Reliability/accuracy |
| Arbelaez Ossa L, Lorenzini G, Milford SR, Shaw D, Elger BS, Rost M. | 2024 | Empirical | AI Generally (no specific method used) | Other/Non-specific | Benefits | Not discussed |
| Arnaout A, Gill P, Virani A, Flatt A, Prodan-Balla N, Byres D, Stowe M, Saremi A, Coss M, Tatto M, Tuason M, Malovec S, Virani S. | 2024 | Conceptual | AI Generally (no specific method used) | Other/Non-specific | Justice (equity or fairness),Bias,Transparency | Not discussed |
| Ast?r?stoae V, Rogozea LM, Lea?u F, Ioan BG. | 2024 | Empirical | AI Generally (no specific method used) | Diagnostic,Clinical monitoring,Treatment | Autonomy (including consent),Benefits,Accountability (ethically responsible for results),Privacy/Confidentiality | Not discussed |
| Baumgartner R, Arora P, Bath C, Burljaev D, Ciereszko K, Custers B, Ding J, Ernst W, Fosch-Villaronga E, Galanos V, Gremsl T, Hendl T, Kropp C, Lenk C, Martin P, Mbelu S, Morais Dos Santos Bruss S, Napiwodzka K, Nowak E, Roxanne T, Samerski S, Schneeberger D, Tampe-Mai K, Vlantoni K, Wiggert K, Williams R. | 2023 | Conceptual | AI Generally (no specific method used) | Diagnostic,Clinical monitoring,Treatment | Justice (equity or fairness),Bias,Transparency | Not discussed |
| Dankwa-Mullan, I | 2024 | Conceptual | AI Generally (no specific method used) | Other/Non-specific | Justice (equity or fairness),Bias,Privacy/Confidentiality | Not discussed |
| Crowe B., Shah S., Teng D. | 2024 | Conceptual | Machine Learning | Diagnostic,Treatment,Other/Non-specific | Autonomy (including consent),Justice (equity or fairness),Bias,Privacy/Confidentiality,Transparency,Other | Clinician acceptability |
| Dumitrascu L., Lespezeanu D., Zugravu C. | 2024 | Empirical | AI Generally (no specific method used) | Screening,Diagnostic,Clinical monitoring,Treatment,Other/Non-specific | Other | Clinician acceptability,Barriers to implementation/adoption within the clinical context,Reliability/accuracy |
| Daneshvar N., Pandita D., Erickson S. | 2024 | Conceptual | Machine Learning | Other/Non-specific | Justice (equity or fairness),Bias,Accountability (ethically responsible for results),Privacy/Confidentiality,Transparency,Other | Not discussed |
| Inglada Galianaa, L. | 2024 | Conceptual | AI Generally (no specific method used) | Other/Non-specific | Autonomy (including consent),Justice (equity or fairness),Bias,Benefits,Accountability (ethically responsible for results),Privacy/Confidentiality,Transparency | Legal liability,Barriers to implementation/adoption within the clinical context,Reliability/accuracy |
| Holtz, B. | 2023 | Conceptual | AI Generally (no specific method used) | Other/Non-specific | Autonomy (including consent),Bias,Benefits,Privacy/Confidentiality | Patient acceptability,Clinician acceptability,Barriers to implementation/adoption within the clinical context |
| Hryciw, B. | 2023 | Conceptual | AI Generally (no specific method used) | Other/Non-specific | Autonomy (including consent),Justice (equity or fairness),Bias,Benefits,Transparency | Patient acceptability,Clinician acceptability,Barriers to implementation/adoption within the clinical context,Reliability/accuracy |
| Huang, Z. | 2024 | Empirical | Other | Diagnostic,Treatment | Autonomy (including consent),Justice (equity or fairness),Bias,Benefits,Accountability (ethically responsible for results),Privacy/Confidentiality,Other | Legal liability,Clinician acceptability,Barriers to implementation/adoption within the clinical context,Reliability/accuracy |
| Hurd, T. | 2024 | Conceptual | AI Generally (no specific method used) | Other/Non-specific | Autonomy (including consent),Justice (equity or fairness),Bias,Accountability (ethically responsible for results),Privacy/Confidentiality,Transparency | Legal liability,Patient acceptability,Clinician acceptability,Barriers to implementation/adoption within the clinical context,Reliability/accuracy |
| Benzinger, L, Ursin, F, Balke WT, Kacprowski T, Salloch S | 2023 | Empirical | AI Generally (no specific method used) | Other/Non-specific | Autonomy (including consent),Justice (equity or fairness),Bias,Benefits,Accountability (ethically responsible for results),Transparency,Other | Clinician acceptability,Barriers to implementation/adoption within the clinical context,Reliability/accuracy |
| Marques, M. et al | 2024 | Empirical | Machine Learning | Diagnostic,Clinical monitoring,Treatment | Justice (equity or fairness),Bias,Accountability (ethically responsible for results),Privacy/Confidentiality,Transparency | Not discussed |
| Mascarenhas M, Afonso J, Ribeiro T, Andrade P, Cardoso H, Macedo G. | 2023 | Empirical | AI Generally (no specific method used) | Diagnostic,Treatment | Bias,Privacy/Confidentiality | Not discussed |
| Khan M, Ewuoso C | 2024 | Conceptual | Other | Other/Non-specific | Autonomy (including consent),Justice (equity or fairness),Bias,Accountability (ethically responsible for results),Transparency | Not discussed |
| Kim JP, Ryan K Kasun M et al | 2023 | Empirical | AI Generally (no specific method used) | Other/Non-specific | Other | Patient acceptability,Clinician acceptability,Barriers to implementation/adoption within the clinical context |
| Kluge EH | 2024 | Conceptual | AI Generally (no specific method used) | Other/Non-specific | Justice (equity or fairness),Bias,Benefits,Privacy/Confidentiality | Not discussed |
| Koranteng E et al | 2023 | Conceptual | Other | Other/Non-specific | Bias | Not discussed |
| Kostick-Quenet, K.; Lang, B.; Smith, J.; Hurley, M.; Blumenthal-Barby, J. | 2024 | Empirical | Machine Learning | Screening,Clinical monitoring | Other | Patient acceptability,Reliability/accuracy |
| Kumar, P.; Chauhan, S.; Awasthi, L. | 2023 | Empirical | AI Generally (no specific method used) | Screening,Diagnostic,Clinical monitoring,Treatment | Bias,Accountability (ethically responsible for results),Privacy/Confidentiality,Transparency | Legal liability,Barriers to implementation/adoption within the clinical context,Reliability/accuracy |
| Mbakwe AB, Lourentzou I, Celi LA, Wu JT. | 2023 | Conceptual | AI Generally (no specific method used) | Diagnostic,Clinical monitoring | Bias | Not discussed |
| Lee, T.; Ho Park, E.; Ho Lee, M. | 2024 | Empirical | AI Generally (no specific method used) | Treatment,Other/Non-specific | Bias | Reliability/accuracy |
| Lewin, S.; Chetty, R.; Ihdayhid, A.; Dwivedi, G. | 2024 | Conceptual | AI Generally (no specific method used) | Diagnostic,Treatment | Autonomy (including consent),Justice (equity or fairness),Bias,Benefits,Accountability (ethically responsible for results),Privacy/Confidentiality,Transparency,Other | Barriers to implementation/adoption within the clinical context,Reliability/accuracy |
| Liebrenz, M.; Bhugra, D.; Alibudbud, R.; Ventriglio, A.; and Smith, A. | 2024 | Conceptual | AI Generally (no specific method used) | Other/Non-specific | Justice (equity or fairness),Bias,Transparency | Not discussed |
| Morrow, E. | 2023 | Empirical | AI Generally (no specific method used) | Other/Non-specific | Autonomy (including consent),Justice (equity or fairness),Benefits | Patient acceptability,Clinician acceptability,Barriers to implementation/adoption within the clinical context |
| Nair, M. | 2024 | Empirical | AI Generally (no specific method used) | Other/Non-specific | Autonomy (including consent),Justice (equity or fairness),Bias,Benefits,Accountability (ethically responsible for results),Privacy/Confidentiality,Transparency,Disclosure of results/Return of results to patients | Legal liability,Patient acceptability,Clinician acceptability,Barriers to implementation/adoption within the clinical context |
| Nelsen, B. | 2023 | Conceptual | AI Generally (no specific method used) | Other/Non-specific | Justice (equity or fairness),Bias,Benefits | Barriers to implementation/adoption within the clinical context,Reliability/accuracy |
| Nichol, A. | 2024 | Empirical | Machine Learning | Other/Non-specific | Bias,Accountability (ethically responsible for results) | Legal liability |
| Nichol, A. | 2023 | Empirical | Machine Learning | Other/Non-specific | Accountability (ethically responsible for results) | Legal liability,Barriers to implementation/adoption within the clinical context |
| ?artolovni, A. | 2023 | Empirical | AI Generally (no specific method used) | Diagnostic,Treatment,Other/Non-specific | Benefits,Accountability (ethically responsible for results) | Barriers to implementation/adoption within the clinical context,Reliability/accuracy |
| Carapinha, J. | 2024 | Conceptual | AI Generally (no specific method used) | Other/Non-specific | Autonomy (including consent),Justice (equity or fairness),Bias,Benefits,Accountability (ethically responsible for results),Privacy/Confidentiality,Transparency,Disclosure of results/Return of results to patients,Other | Reliability/accuracy |
| Collins, B. | 2024 | Empirical | AI Generally (no specific method used) | Other/Non-specific | Justice (equity or fairness),Bias,Privacy/Confidentiality | Barriers to implementation/adoption within the clinical context,Reliability/accuracy |
| Bouhouita??uermech, S, Haidar, H | 2024 | Empirical | AI Generally (no specific method used) | Other/Non-specific | Autonomy (including consent),Justice (equity or fairness),Accountability (ethically responsible for results),Privacy/Confidentiality,Transparency | Legal liability,Reliability/accuracy |
| Petersen L et al | 2023 | Empirical | AI Generally (no specific method used) | Other/Non-specific | Autonomy (including consent),Justice (equity or fairness),Benefits,Transparency,Other | Barriers to implementation/adoption within the clinical context |
| Cagliero, D, Deuitch, N, Shah, N, et al. | 2023 | Empirical | Machine Learning | Other/Non-specific | Autonomy (including consent),Justice (equity or fairness),Bias,Benefits,Transparency,Disclosure of results/Return of results to patients,Other | Barriers to implementation/adoption within the clinical context,Reliability/accuracy |
| Ciro M., Umberto M., Giuseppe DP., & Massimo E. | 2024 | Conceptual | AI Generally (no specific method used) | Screening,Diagnostic,Clinical monitoring,Treatment,Other/Non-specific | Autonomy (including consent),Justice (equity or fairness),Benefits,Accountability (ethically responsible for results),Privacy/Confidentiality,Transparency | Patient acceptability,Clinician acceptability,Reliability/accuracy |
| Mirzaei1 T., Amini L., and Esmaeilzadeh P. | 2024 | Empirical | Machine Learning | Screening,Diagnostic,Clinical monitoring,Treatment | Autonomy (including consent),Justice (equity or fairness),Bias,Privacy/Confidentiality,Transparency | Not discussed |
| Moodley K. | 2024 | Conceptual | AI Generally (no specific method used) | Other/Non-specific | Autonomy (including consent),Justice (equity or fairness),Bias,Accountability (ethically responsible for results),Privacy/Confidentiality,Transparency | Not discussed |
| Mooghali1 M., Stroud A., Yoo D., Barry B., Grimshaw A., Ross J., Zhu X., and Miller J. | 2024 | Empirical | AI Generally (no specific method used) | Other/Non-specific | Autonomy (including consent),Bias,Accountability (ethically responsible for results),Privacy/Confidentiality,Transparency | Patient acceptability,Clinician acceptability |
| Morris M., Song E., Rajesh A., Asaad M., and Phillips B. | 2023 | Conceptual | AI Generally (no specific method used) | Other/Non-specific | Autonomy (including consent),Bias,Benefits,Privacy/Confidentiality | Legal liability,Reliability/accuracy |
| Post B et al | 2022 | Conceptual | AI Generally (no specific method used) | Other/Non-specific | Benefits | Not discussed |
| Kahraman, F. | 2024 | Empirical | AI Generally (no specific method used) | Other/Non-specific | Autonomy (including consent),Justice (equity or fairness),Benefits,Accountability (ethically responsible for results),Privacy/Confidentiality,Other | Barriers to implementation/adoption within the clinical context,Reliability/accuracy |
| Kasun, M. | 2023 | Empirical | Machine Learning | Other/Non-specific | Bias,Accountability (ethically responsible for results),Transparency,Other | Clinician acceptability,Barriers to implementation/adoption within the clinical context,Reliability/accuracy |
| Katirai, A. | 2023 | Conceptual | AI Generally (no specific method used) | Screening | Autonomy (including consent),Justice (equity or fairness),Bias,Benefits,Accountability (ethically responsible for results),Privacy/Confidentiality,Transparency,Other | Reliability/accuracy |
| Katwaroo, A. | 2024 | Conceptual | AI Generally (no specific method used) | Diagnostic,Clinical monitoring,Treatment,Other/Non-specific | Autonomy (including consent),Justice (equity or fairness),Bias,Benefits,Accountability (ethically responsible for results),Privacy/Confidentiality,Transparency | Barriers to implementation/adoption within the clinical context,Reliability/accuracy |
| Wang C et al | 2023 | Conceptual | AI Generally (no specific method used) | Other/Non-specific | Justice (equity or fairness),Bias,Benefits,Accountability (ethically responsible for results),Privacy/Confidentiality,Transparency,Disclosure of results/Return of results to patients,Other | Legal liability,Reliability/accuracy |
| Wang W et al | 2024 | Empirical | AI Generally (no specific method used) | Other/Non-specific | Autonomy (including consent),Justice (equity or fairness),Bias,Accountability (ethically responsible for results),Privacy/Confidentiality,Transparency | Clinician acceptability,Barriers to implementation/adoption within the clinical context,Reliability/accuracy |
| Stevens Y., & Zawati M. | 2024 | Conceptual | Machine Learning | Diagnostic,Treatment | Autonomy (including consent),Accountability (ethically responsible for results),Transparency,Other | Legal liability,Patient acceptability,Clinician acceptability,Barriers to implementation/adoption within the clinical context,Reliability/accuracy |
| Sung, J. | 2023 | Conceptual | AI Generally (no specific method used) | Screening,Diagnostic,Clinical monitoring,Treatment | Autonomy (including consent),Accountability (ethically responsible for results),Other | Legal liability,Patient acceptability,Barriers to implementation/adoption within the clinical context,Reliability/accuracy |
| Tang L., Li J., and Fantus S. | 2023 | Empirical | AI Generally (no specific method used) | Screening,Diagnostic | Autonomy (including consent),Justice (equity or fairness),Bias,Benefits,Accountability (ethically responsible for results),Privacy/Confidentiality,Transparency | Barriers to implementation/adoption within the clinical context |
| Tavory, T. | 2024 | Conceptual | AI Generally (no specific method used) | Other/Non-specific | Autonomy (including consent),Justice (equity or fairness),Bias,Accountability (ethically responsible for results),Privacy/Confidentiality,Transparency | Legal liability,Reliability/accuracy |
| Wabro A et al | 2024 | Conceptual | AI Generally (no specific method used) | Other/Non-specific | Benefits | Not discussed |
| Theriault-Lauzier P., Cobin D., Tastet O. | 2024 | Conceptual | AI Generally (no specific method used) | Other/Non-specific | Bias | Not discussed |
| Reddy, S. | 2023 | Conceptual | AI Generally (no specific method used) | Other/Non-specific | Bias,Benefits,Accountability (ethically responsible for results),Privacy/Confidentiality,Transparency | Legal liability,Reliability/accuracy |
| Pozzi G. | 2023 | Conceptual | Machine Learning | Screening,Diagnostic | Justice (equity or fairness),Bias,Transparency | Not discussed |
| Pressman SM, Borna S, Gomez-Cabello CA, Haider SA, Haider C, Forte AJ. | 2024 | Empirical | AI Generally (no specific method used) | Diagnostic,Treatment | Autonomy (including consent),Bias,Accountability (ethically responsible for results),Privacy/Confidentiality | Reliability/accuracy |
| Prochaska M, Alfandre D. | 2024 | Conceptual | AI Generally (no specific method used) | Screening,Diagnostic,Clinical monitoring,Treatment | Autonomy (including consent),Accountability (ethically responsible for results) | Not discussed |
| Rose, S. | 2024 | Conceptual | AI Generally (no specific method used) | Other/Non-specific | Autonomy (including consent),Accountability (ethically responsible for results),Transparency,Disclosure of results/Return of results to patients | Patient acceptability,Barriers to implementation/adoption within the clinical context |
| Sacca, R. | 2024 | Empirical | AI Generally (no specific method used) | Other/Non-specific | Autonomy (including consent),Bias,Transparency | Patient acceptability |
| Savulescu, J. | 2024 | Conceptual | AI Generally (no specific method used) | Other/Non-specific | Autonomy (including consent),Justice (equity or fairness),Bias,Benefits,Accountability (ethically responsible for results),Privacy/Confidentiality,Transparency | Legal liability,Barriers to implementation/adoption within the clinical context,Reliability/accuracy |
| Pruski M. | 2024 | Conceptual | AI Generally (no specific method used) | Diagnostic,Clinical monitoring,Treatment | Autonomy (including consent),Benefits,Privacy/Confidentiality | Reliability/accuracy |
| Wang Y, Song Y et al | 2023 | Conceptual | AI Generally (no specific method used) | Other/Non-specific | Justice (equity or fairness),Bias | Not discussed |
| Witkowski K et al | 2024 | Empirical | AI Generally (no specific method used) | Other/Non-specific | Benefits | Patient acceptability |
| Weidener L and Fischer M | 2024 | Empirical | AI Generally (no specific method used) | Other/Non-specific | Justice (equity or fairness),Bias | Not discussed |
| Webb J | 2024 | Conceptual | AI Generally (no specific method used) | Other/Non-specific | Autonomy (including consent),Transparency,Other | Not discussed |
| Sisk, B.; Antes, A.; DuBois, J. | 2024 | Conceptual | Machine Learning | Diagnostic,Clinical monitoring,Other/Non-specific | Autonomy (including consent),Justice (equity or fairness),Privacy/Confidentiality,Transparency | Reliability/accuracy |
| Skuban-Eiseler, T.; Orzechowski, M.; Denkinger, M.; Kocar, T.; Leinert, C.; Steger, F. | 2023 | Empirical | AI Generally (no specific method used) | Clinical monitoring,Treatment | Autonomy (including consent),Justice (equity or fairness),Transparency | Not discussed |
| Smith, H. | 2024 | Conceptual | AI Generally (no specific method used) | Clinical monitoring,Treatment,Other/Non-specific | Accountability (ethically responsible for results) | Legal liability,Reliability/accuracy |
| Smith, H.; Downer, J.; Ives, J. | 2023 | Conceptual | AI Generally (no specific method used) | Diagnostic,Clinical monitoring,Treatment,Other/Non-specific | Justice (equity or fairness),Accountability (ethically responsible for results),Transparency | Legal liability,Reliability/accuracy |
| Tiribelli, S. | 2024 | Conceptual | AI Generally (no specific method used) | Other/Non-specific | Autonomy (including consent),Justice (equity or fairness),Bias,Benefits,Privacy/Confidentiality,Transparency | Reliability/accuracy |
| Tursynbek1, A. | 2024 | Empirical | AI Generally (no specific method used) | Other/Non-specific | Autonomy (including consent),Bias,Benefits,Accountability (ethically responsible for results),Privacy/Confidentiality | Legal liability,Reliability/accuracy |
| ?L?KHAN1, S. | 2024 | Conceptual | AI Generally (no specific method used) | Diagnostic,Treatment | Justice (equity or fairness),Bias,Benefits,Accountability (ethically responsible for results),Privacy/Confidentiality,Transparency | Reliability/accuracy |
| Smith, A.; Arena, R.; Bacon, S.; et al. | 2024 | Conceptual | AI Generally (no specific method used) | Clinical monitoring,Other/Non-specific | Autonomy (including consent),Justice (equity or fairness),Bias,Transparency | Patient acceptability,Reliability/accuracy |
| Nichol, A; Sankar, P; Halley, M; et al | 2023 | Empirical | AI Generally (no specific method used) | Other/Non-specific | Bias,Accountability (ethically responsible for results),Privacy/Confidentiality,Transparency,Other | Legal liability,Reliability/accuracy |
| Ning, Y; Teixayavong, S; Shang, Y; et al | 2024 | Empirical | Other | Other/Non-specific | Autonomy (including consent),Justice (equity or fairness),Accountability (ethically responsible for results),Privacy/Confidentiality,Transparency | Legal liability,Clinician acceptability,Reliability/accuracy |
| Hatherley J | 2024 | Conceptual | AI Generally (no specific method used) | Other/Non-specific | Autonomy (including consent),Justice (equity or fairness),Bias,Transparency,Disclosure of results/Return of results to patients | Not discussed |
| Kim B et al | 2024 | Empirical | AI Generally (no specific method used) | Screening,Diagnostic | Autonomy (including consent) | Patient acceptability,Barriers to implementation/adoption within the clinical context |
| Pruski M | 2024 | Conceptual | AI Generally (no specific method used) | Other/Non-specific | Justice (equity or fairness) | Legal liability |
| Meier, E., Rigter, T., Schijven, M., Van den Hoven, M., Bak., M. | 2024 | Empirical | Other | Other/Non-specific | Accountability (ethically responsible for results) | Not discussed |
| Palianiappan, K; Lin, E; Vogel, S; Lim, J | 2024 | Empirical | AI Generally (no specific method used) | Other/Non-specific | Autonomy (including consent),Justice (equity or fairness),Bias,Accountability (ethically responsible for results),Privacy/Confidentiality,Transparency | Legal liability,Clinician acceptability,Reliability/accuracy |
| Parchmann, N; Hansen, D; Orzechowski, M; Steger, F | 2024 | Empirical | AI Generally (no specific method used) | Diagnostic,Treatment | Autonomy (including consent),Justice (equity or fairness),Bias,Benefits,Accountability (ethically responsible for results),Privacy/Confidentiality,Transparency | Patient acceptability,Clinician acceptability,Reliability/accuracy |
| Lee WT. | 2024 | Conceptual | AI Generally (no specific method used) | Diagnostic | Accountability (ethically responsible for results) | Not discussed |
| Montomoli J, Bitondo MM, Cascella M, Rezoagli E, Romeo L, Bellini V, Semeraro F, Gamberini E, Frontoni E, Agnoletti V, Altini M, Benanti P, Bignami EG. | 2024 | Conceptual | AI Generally (no specific method used) | Diagnostic | Bias,Transparency | Not discussed |
| Espejo G, Reiner W, Wenzinger M. | 2023 | Conceptual | AI Generally (no specific method used) | Diagnostic,Clinical monitoring | Justice (equity or fairness),Accountability (ethically responsible for results),Privacy/Confidentiality | Not discussed |
| Vandemeulebroucke, T. | 2024 | Conceptual | AI Generally (no specific method used) | Diagnostic,Clinical monitoring,Treatment,Other/Non-specific | Autonomy (including consent),Justice (equity or fairness),Bias,Benefits,Accountability (ethically responsible for results),Privacy/Confidentiality,Transparency | Reliability/accuracy |
| Khan, S. | 2024 | Conceptual | AI Generally (no specific method used) | Other/Non-specific | Autonomy (including consent),Justice (equity or fairness),Bias,Benefits,Accountability (ethically responsible for results),Privacy/Confidentiality,Transparency | Legal liability,Patient acceptability,Clinician acceptability,Reliability/accuracy |
| Wu, C. | 2023 | Empirical | AI Generally (no specific method used) | Other/Non-specific | Autonomy (including consent),Justice (equity or fairness),Bias,Accountability (ethically responsible for results),Privacy/Confidentiality,Transparency | Legal liability,Patient acceptability,Barriers to implementation/adoption within the clinical context |
| Zhang, J. | 2023 | Conceptual | AI Generally (no specific method used) | Other/Non-specific | Autonomy (including consent),Justice (equity or fairness),Bias,Benefits,Accountability (ethically responsible for results),Privacy/Confidentiality,Transparency | Legal liability,Patient acceptability,Clinician acceptability,Reliability/accuracy |
| Abbasian, M; Khatibi, E; Azimi, I; et al. | 2024 | Conceptual | Other | Other/Non-specific | Justice (equity or fairness),Bias,Privacy/Confidentiality,Transparency,Other | Reliability/accuracy |
| Adams, J | 2023 | Conceptual | AI Generally (no specific method used) | Other/Non-specific | Accountability (ethically responsible for results),Transparency,Other | Legal liability |
| Yelne, S. | 2023 | Conceptual | AI Generally (no specific method used) | Other/Non-specific | Autonomy (including consent),Justice (equity or fairness),Bias,Accountability (ethically responsible for results),Privacy/Confidentiality,Transparency | Legal liability,Barriers to implementation/adoption within the clinical context,Reliability/accuracy |
| Zawati, M. | 2024 | Conceptual | Other | Diagnostic | Autonomy (including consent),Justice (equity or fairness),Bias,Accountability (ethically responsible for results) | Legal liability,Clinician acceptability,Reliability/accuracy |
| Vandersluis, R. | 2024 | Empirical | AI Generally (no specific method used) | Diagnostic,Other/Non-specific | Justice (equity or fairness),Bias,Benefits,Transparency | Reliability/accuracy |
| Al-Ani, A; Rayyan, A; Maswadeh, A; et al. | 2024 | Empirical | AI Generally (no specific method used) | Other/Non-specific | Autonomy (including consent),Bias,Accountability (ethically responsible for results),Privacy/Confidentiality | Not discussed |
| Aquino, Y; Carter, S; Houssami, N; et al. | 2023 | Empirical | AI Generally (no specific method used) | Screening,Diagnostic,Other/Non-specific | Justice (equity or fairness),Bias,Accountability (ethically responsible for results),Transparency | Legal liability,Reliability/accuracy |
| Ahmad, M; Eckert, C | 2023 | Conceptual | AI Generally (no specific method used) | Screening,Diagnostic,Other/Non-specific | Justice (equity or fairness),Bias,Transparency | Barriers to implementation/adoption within the clinical context,Reliability/accuracy |
| Maccaro A, Stokes K, Statham L, He L, Williams A, Pecchia L, Piaggio D. | 2024 | Empirical | AI Generally (no specific method used) | Other/Non-specific | Autonomy (including consent),Justice (equity or fairness),Accountability (ethically responsible for results),Privacy/Confidentiality,Transparency | Not discussed |
| MacIntyre MR, Cockerill RG, Mirza OF, Appel JM. | 2023 | Conceptual | AI Generally (no specific method used) | Screening,Diagnostic | Autonomy (including consent),Bias,Accountability (ethically responsible for results) | Not discussed |
| Tiribelli, S.; Monnot, A.; Shah, S.; Arora, A.; Toong, P.; Kong, S. | 2023 | Conceptual | Machine Learning | Screening,Diagnostic,Clinical monitoring | Autonomy (including consent),Justice (equity or fairness),Bias,Benefits,Accountability (ethically responsible for results),Privacy/Confidentiality,Transparency | Legal liability,Patient acceptability,Barriers to implementation/adoption within the clinical context,Reliability/accuracy |
| Altamimi I., Altamimi A., Alhumimidi A., Altamimi A., Temsah M. | 2023 | Conceptual | AI Generally (no specific method used) | Diagnostic,Treatment | Autonomy (including consent),Accountability (ethically responsible for results),Privacy/Confidentiality,Transparency | Not discussed |
| Badawy, W., Zinhom, H., & Shaban, M. | 2024 | Empirical | AI Generally (no specific method used) | Other/Non-specific | Autonomy (including consent),Justice (equity or fairness),Bias,Accountability (ethically responsible for results),Privacy/Confidentiality | Legal liability |
| Lukkien D, Stolwijk N, Ipakchian Askari S, et al. | 2024 | Empirical | Other | Clinical monitoring | Autonomy (including consent),Justice (equity or fairness),Bias,Benefits,Privacy/Confidentiality,Transparency | Patient acceptability,Clinician acceptability,Reliability/accuracy |
| Ossa, L; Milford, S; Rost, M; et al. | 2024 | Empirical | AI Generally (no specific method used) | Other/Non-specific | Autonomy (including consent),Benefits,Privacy/Confidentiality,Transparency,Other | Not discussed |
| Vo V, Chen G, Saint James Aquino Y, | 2023 | Empirical | AI Generally (no specific method used) | Screening,Diagnostic,Clinical monitoring,Treatment,Other/Non-specific | Autonomy (including consent),Justice (equity or fairness),Bias,Benefits,Accountability (ethically responsible for results),Privacy/Confidentiality,Transparency | Legal liability,Patient acceptability,Clinician acceptability,Barriers to implementation/adoption within the clinical context,Reliability/accuracy |
| Lukkien D, Ipakchian Askari S, Stolwijk N, et al. | 2024 | Empirical | Other | Clinical monitoring | Autonomy (including consent),Bias,Benefits,Accountability (ethically responsible for results),Transparency | Not discussed |
| Ranjbar, A, Skolt, K, Theodore A, Aakenes, KT, et al. | 2023 | Empirical | AI Generally (no specific method used) | Other/Non-specific | Bias | Not discussed |
